# Experimental warming increases fungal alpha diversity in an oligotrophic maritime Antarctic soil

**DOI:** 10.3389/fmicb.2022.1050372

**Published:** 2022-11-10

**Authors:** Kevin K. Newsham, Marta Misiak, William P. Goodall-Copestake, Malin Stapnes Dahl, Lynne Boddy, David W. Hopkins, Marie L. Davey

**Affiliations:** ^1^British Antarctic Survey, NERC, Cambridge, United Kingdom; ^2^School of Biosciences, Cardiff University, Cardiff, United Kingdom; ^3^The Scottish Association for Marine Science, Oban, United Kingdom; ^4^Department of Biology, University of Oslo, Oslo, Norway; ^5^Scotland’s Rural College, Edinburgh, United Kingdom; ^6^Norwegian Institute for Nature Research, Trondheim, Norway

**Keywords:** Antarctica, climate warming, open top chambers (OTCs), organic carbon, organic nitrogen, soil fungal community diversity, yeasts

## Abstract

The climate of maritime Antarctica has altered since the 1950s. However, the effects of increased temperature, precipitation and organic carbon and nitrogen availability on the fungal communities inhabiting the barren and oligotrophic fellfield soils that are widespread across the region are poorly understood. Here, we test how warming with open top chambers (OTCs), irrigation and the organic substrates glucose, glycine and tryptone soy broth (TSB) influence a fungal community inhabiting an oligotrophic maritime Antarctic fellfield soil. In contrast with studies in vegetated soils at lower latitudes, OTCs increased fungal community alpha diversity (Simpson’s index and evenness) by 102–142% in unamended soil after 5 years. Conversely, OTCs had few effects on diversity in substrate-amended soils, with their only main effects, in glycine-amended soils, being attributable to an abundance of *Pseudogymnoascus*. The substrates reduced alpha and beta diversity metrics by 18–63%, altered community composition and elevated soil fungal DNA concentrations by 1–2 orders of magnitude after 5 years. In glycine-amended soil, OTCs decreased DNA concentrations by 57% and increased the relative abundance of the yeast *Vishniacozyma* by 45-fold. The relative abundance of the yeast *Gelidatrema* declined by 78% in chambered soil and increased by 1.9-fold in irrigated soil. Fungal DNA concentrations were also halved by irrigation in TSB-amended soils. In support of regional- and continental-scale studies across climatic gradients, the observations indicate that soil fungal alpha diversity in maritime Antarctica will increase as the region warms, but suggest that the accumulation of organic carbon and nitrogen compounds in fellfield soils arising from expanding plant populations are likely, in time, to attenuate the positive effects of warming on diversity.

## Introduction

Maritime Antarctica has warmed in recent decades, with rises in mean annual surface air temperature of up to 0.5°C per decade having been recorded across the region since the 1950s (Adams et al., 2009). Warming has led to profound changes to the physical environment of the region, including widespread glacial recession, ice shelf disintegration and more frequent precipitation events (Turner et al., 1997; Fox and Cooper, 1998; Vaughan et al., 2003; Cook et al., 2005). In agreement with the current hiatus in warming at the global scale (Kosaka and Xie, 2013), analyses of temperature trends indicate a cessation of warming in the region since around the turn of the millennium (Turner et al., 2016). However, modelling studies predict that warming will resume in Antarctica over future decades. Under uncontrolled emissions of greenhouse gases, increases in mean annual surface air temperatures of up to 4.8°C, accompanied by a 24% increase in the frequency of precipitation events, are anticipated across the continent by 2100 (Bracegirdle et al., 2020). Assuming a stabilisation of greenhouse gas emissions, however, studies predict mean annual surface temperatures to rise by 2.5°C and precipitation events to increase in frequency by approximately 16% in Antarctica by the end of the 21st century (Bracegirdle et al., 2020).

Warming, combined with more frequent precipitation and the exposure of new terrain in front of receding glaciers (Lee et al., 2017), has led in recent decades to the expansion of maritime Antarctic plant populations into the barren fellfield soils that are widespread across the region (Fowbert and Smith, 1994; Royles et al., 2013; Cannone et al., 2022). As a consequence, rhizodeposition and litter inputs will have elevated the concentrations of sugars, amino acids, peptides and proteins in these soils (Jones et al., 2004; Yergeau et al., 2007; Strauss et al., 2009). Nevertheless, despite the potential for increasing temperatures, precipitation and organic C and N concentrations to affect the diversity and abundance of soil-dwelling microbes, the responses of maritime Antarctic soil fungal communities to these factors are poorly understood. This represents a considerable gap in current knowledge, since fungi have pivotal roles in all soils as decomposers of organic matter (Swift et al., 1979) and as partners in the lichen symbiosis, which is frequent in maritime Antarctica (Øvstedal and Smith, 2001). Of the few studies to have predicted the effects of climate change on soil fungal communities in the region, a space-for-time substitution study of fellfield soils sampled from across a 1,650 km climatic gradient showed a positive association between operational taxonomic unit (OTU) richness and surface air temperature, but could not resolve whether higher OTU richness at lower latitudes is associated with increased temperature or liquid water availability (Newsham et al., 2016). Studies based on single samplings of maritime Antarctic soils warmed with open top chambers (OTCs) for 3–8 years have also shown 20–30% increases in the frequencies of fungal 18S rRNA gene copies and the concentrations of phospholipid fatty acids in soil, but no apparent effects on fungal OTU richness or alpha diversity metrics (Yergeau et al., 2012; Kim et al., 2018).

Despite the majority of maritime Antarctic soils being devoid of vegetation (Bockheim, 2015), previous studies in the region have deployed OTCs on soils with dense to sparse plant or lichen cover (Yergeau et al., 2007, 2012; Kim et al., 2018). Here, to more accurately predict the responses of maritime Antarctic soils to climate change, we test the main and interaction effects of warming with OTCs, irrigation and organic C and N inputs across several samplings of a field experiment on the diversity and size of a fungal community inhabiting an oligotrophic fellfield soil. Based on observations from previous studies using OTCs in maritime Antarctica, we anticipated that warming would increase soil fungal DNA concentrations but would have few effects on fungal community alpha diversity (Yergeau et al., 2012; Kim et al., 2018). In addition, we expected that warming combined with organic C and N inputs would alter fungal DNA concentrations in soil (Misiak et al., 2021). Given the strong effects of vegetation cover in polar soils on fungal diversity and abundance – with vegetated soils hosting fewer fungal species and lower numbers of unique taxa than fellfield soils (Grau et al., 2017; Canini et al., 2019) – we also anticipated different responses of the fungal community to warming than those recorded in densely vegetated soils at lower latitudes.

## Materials and methods

### Field experiment

**FIGURE 1:**
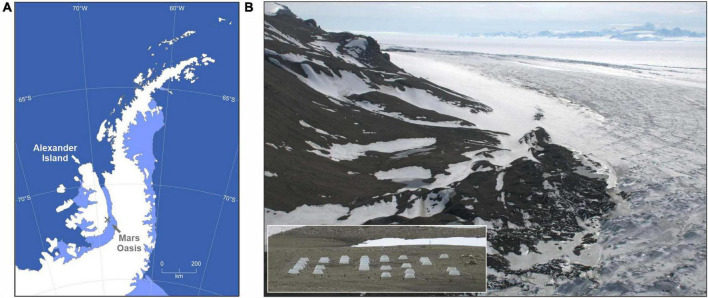
Mars Oasis in the southern maritime Antarctic. **(A)** Map of the Antarctic Peninsula, showing the location of Mars Oasis on Alexander Island and **(B)** an aerial view of the oasis, showing the periglacial landscape and George VI Ice Shelf to the rear. Inset in panel **(B)** shows the field experiment, consisting of 64 plots, 32 of which were covered with open top chambers.

The experiment was deployed in November 2007 at Mars Oasis on south-eastern Alexander Island (Figure 1A). Higher animals, including nesting birds and seals, are absent from the site. A full description of the experiment is provided by Misiak et al. (2021). Briefly, it consisted of 64 plots of 1 m diameter on moraine fellfield soil in a periglacial habitat (Figure 1B). The soil, which is devoid of vegetation and underlain by ice-cemented permafrost at 100–300 mm depth (Sugden and Clapperton, 1981), is permanently frozen for *c*. 39 weeks each year and is free of snow cover for *c*. 10 weeks in early December–mid February. Annual minimum, mean and maximum temperatures of unchambered soil at 25–30 mm depth are −32, −7, and 19°C (Misiak et al., 2021). The oligotrophic soil has mean organic C and N concentrations of 0.26% and 0.02%, respectively, an average pH value of 7.9, and is arid, with water potential declining to −7 MPa within a month of snowmelt (Hopkins et al., 2021; Misiak et al., 2021). Precipitation at Mars Oasis is extremely sparse, with approximately one snow- or rainfall event occurring each year between December and mid February (Misiak et al., 2021).

Soil in 48 plots was amended annually with either glucose, glycine (the most frequent amino acid in maritime Antarctic soils; Hill et al., 2019) or tryptone soy broth (TSB, a mixture of amino acids, peptides and proteins). Soil in 16 plots received each of the three substrates, which were applied by mixing in powder to *c*. 30 mm depth with sterile spoons. The substrates elevated soil C concentrations to 0.39% (glucose), 0.36% (glycine and TSB) and soil N concentrations to approximately 0.08% (glycine) and 0.05% (TSB). Soil in 16 plots did not receive substrates, but was also mixed to *c*. 30 mm depth with sterile spoons. Within each group of 16 plots, eight were warmed for the duration of the experiment with OTCs (Figure 1B, inset), which increased mean soil temperature at 25–30 mm depth by 1.7°C during December and January, and eight were irrigated annually with 1.5 L of deionized water applied to a 0.05 m^2^ central area (Misiak et al., 2021). The irrigation treatment, which raised soil moisture to 100% of water holding capacity, approximated to 30 mm water equivalent, or, assuming a bulk density for Antarctic surface snow of 0.35 g cm^–3^ (Van den Broeke, 2008), snowcover of 85 mm depth melting over soil. The experimental layout resulted in 16 OTC-irrigation-substrate treatments, each replicated four times in a randomised design (Supplementary Figure 1). Soil samples were collected in early December 2007, 2009, 2010, 2011, and 2012 and were transported at −20°C to the UK, where DNA was extracted from them as described by Misiak et al. (2021).

### ITS1 metabarcoding

The fungal community in the 320 soil samples was characterized by metabarcoding the internal transcribed spacer 1 region of nuclear ribosomal DNA. Despite the potential for long insertions in this region, previous studies indicate that it yields similar community composition data to those from the internal transcribed spacer 2 region when used as a DNA metabarcode for fungi (Bazzicalupo et al., 2013; Blaalid et al., 2013). Ribosomal DNA between the 3′ end of the 18S gene and the 5′ end of the 28S gene was initially amplified from the DNA extracts with the fungal-specific primers ITS1F (5′-CTTGGTCATTTAGAGGAGTAA-3′; Gardes and Bruns, 1993) and ITS4 (5′-TCCTCCGCTTATTGATATGC-3′; White et al., 1990). The internal transcribed spacer 1 region was then tagged in a second, nested PCR using the fusion primers ITS1F and ITS2 (5′-GCTGCGTTCTTCATCGATGC-3′; White et al., 1990) and one of 96 unique six basepair (bp) multiple identifier combinations. PCR reactions were conducted in 25 μL reactions, with final concentrations of reagents as follows: 1 × Gold buffer, 0.2 mM dNTPs, 1.5 mM MgCl_2_, 0.2 μM each of the forward and reverse primers, 2 μL of template DNA and 0.75 units of AmpliTaq Gold^®^ DNA Polymerase (ThermoFisher Scientific, Waltham, MA, USA). Reaction conditions consisted of initial denaturation at 95°C for 5 min followed by 30 cycles (initial amplification) or 20 cycles (nested amplification) of denaturation at 95°C for 30 s, annealing at 55°C for 30 s, elongation at 72°C for 1 min and a final elongation step of 72°C for 7 min. Amplicons were purified with Agencourt AMPure beads (Agencourt Bioscience, Beverly, United States) and DNA concentrations were measured with Qubit assays. Samples were combined into equimolar pools of 96 uniquely tagged samples that were submitted to StarSeq GmbH (Mainz, Germany), where each of the four pools of 96 samples were further indexed (Illumina Nextera Library Prep kit) before sequencing in a paired end (300 bp × 2) run on an Illumina MiSeq platform (Illumina Inc., San Diego, CA, USA).

Samples were demultiplexed and primers removed from the 5′ and 3′ ends of forward and reverse reads using cutadapt v.1.9.1 (Martin, 2011). Successful demultiplexing required complete overlap of the multiple identifier and primer sequence length with no expected errors. The DADA2 v1.18 package in R (Callahan et al., 2016) was used for quality filtering, error correction and chimera detection. Reads were quality filtered to remove all sequences with ambiguous bases and >2 or >5 expected errors in the forward and reverse directions, respectively. Error rates were estimated for forward and reverse sequences, which were subsequently merged with a minimum overlap of 30 bp, and amplicon sequence variants were inferred for each sample. Chimeric sequence variants were assessed on a per-sample basis, as chimeric events occur at the individual PCR level. Sequence variants were removed if they were flagged as chimeric in >90% of samples in which they occurred. Amplicon sequence variants were further clustered into OTUs at 97% similarity using VSEARCH v. 2.14.1 (Rognes et al., 2016) and the most abundant sequence variant was selected as the representative sequence of the OTU.

Taxonomy was assigned to OTUs using the RDP classifier (Wang et al., 2007) against the UNITE database (Nilsson et al., 2018). In cases where the RDP classifier assigned OTUs to genus level with a probability of at least 80%, OTUs were further assigned to a guild or growth form (lichenised, obligate and facultative yeasts, or filamentous) using FUNguild v. 1.0 (Nguyen et al., 2016) and previous data (Newsham et al., 2021), including only the taxa from the former for which the guild assignments were “highly probable” or “probable.” In addition, OTUs assigned to the Verrucariaceae, Teloschistaceae, Lecanoraceae and Acarosporaceae at a probability of at least 80% were assigned to the lichenised guild, since these families consist entirely of lichen-forming taxa. Thirty-two samples, consisting of 16, 14 and two soils from 2007, 2010, and 2011, respectively, generated <5,000 sequences, and were hence removed from further analyses. The mean richness estimate for the seven samples of unamended soils from 2010 that remained following the removal of these samples was two-thirds lower than that for unamended soils sampled in other years (Supplementary Figure 2), and these seven samples were therefore also removed from further analyses.

### Quantitative-PCR assays

The weight of fungal DNA in soil was determined using quantitative (Q)-PCR using previously described methods (Misiak et al., 2021), but with the fungal-specific primers ITS86F (5′-GTGAATCATCGAATCTTTGAA-3′; Turenne et al., 1999) and ITS4 (Op De Beeck et al., 2014). A calibration curve was produced using a dilution series of DNA (range 4.64 × 10^–5^–46.4 ng DNA μl^–1^) amplified from Mars Oasis soil with the ITS86F/ITS4 primer pair, with values subsequently being converted to ng DNA g^–1^ dwt (48 h at 105°C) soil. Melting curves indicated that single products were amplified during PCRs.

### Statistical analyses

Community alpha diversity metrics, *viz*., Simpson’s diversity index, Simpson’s evenness and OTU richness, were calculated using the *iNEXT* package in R (Hsieh et al., 2016). The vegan package in R (Oksanen et al., 2019) was used to calculate community beta diversity metrics, *viz*., year-to-year community turnover (Bray-Curtis dissimilarity) and within-group variance in community composition (distance to centroid values). Values were averaged per plot over 2009–2012 and were tested for normality using Kolmogorov–Smirnov tests. Where necessary, values were log_10_-transformed prior to analyses using general linear models (GLMs) in MINITAB 19 (State College, PA, USA), testing for the main and interaction effects of OTCs and irrigation in unamended, glucose-, glycine- or TSB-amended soils. Principal component analysis (PCA) was also used to determine associations between the relative abundances of the 30 most frequent taxa and the treatments. PERMANOVA and variance partitioning analyses were conducted in the vegan package to examine the effects of year and treatments on beta diversity. To detect when substrate, OTC and irrigation effects first occurred, and to determine the magnitudes of the effects, PERMANOVA analyses were conducted on data from each sampling between 2009 and 2012. Variance partitioning was also used to identify the relative contributions of year and treatments to the observed differences in community composition. Relative abundances of taxa and DNA concentration values averaged per plot over 2009–2012 consistently failed Kolmogorov–Smirnov tests, and so were analyzed in SPSS (IBM) using GLMs with factorial contrasts and bootstrapping (10,000 randomizations) of log_10_-transformed data (Misiak et al., 2021). Bonferroni correction was applied to the analyses on each taxon, with the alpha threshold value for treatment effects being reduced to 0.0017. To identify taxa representative of each treatment in 2009–2012, indicator species analysis in R (De Cáceres and Legendre, 2009) was conducted on taxa with minimum occupancies of seven in the dataset, with factorial contrasts subsequently being used to determine treatment effects on frequent taxa.

## Results

### Community composition

A total of 415 fungal OTUs were recorded, with OTU accumulation curves for the majority of samples closely approaching an asymptote (Supplementary Figure 2). Thirty frequent OTUs accounted for >90% of the community across all samplings and treatments (Table 1). The most abundant OTU was a species of *Pseudogymnoascus*, the only frequent filamentous fungus that could be confidently identified to genus level (Table 1). Thirty seven infrequent OTUs belonging to genera with obligate filamentous growth forms were also recorded. Yeasts were the most abundant growth form, with 58 OTUs being classified as either obligate or facultative yeasts, and with a member of the Microbotryomycetes, species of *Vishniacozyma*, *Gelidatrema*, *Leucosporidium* and *Rhinocladiella*, and *Vishniacozyma victoriae*, *V. tephrensis*, *Mrakia frigida* and *Naganishia friedmannii* being frequent in the community (Table 1). Lichen-forming fungi were less abundant, with 38 OTUs being classified as lichenised forms, and with *Austroplaca darbishirei* and *Polyblastia bryophila* being frequent in the community (Table 1). Mycorrhizal symbionts were not recorded in soil.

**TABLE 1:** The 30 operational taxonomic units (OTUs) accounting for >90% of the soil fungal community at Mars Oasis.

OTU number	Identity	Growth form or guild	Classification probability (%)*
0	*Pseudogymnoascus* sp.	Filamentous	100
1	Eurotiomycete	Uncl.	81
10	*Naganishia friedmannii*	Yeast	95
100	*Austroplaca darbishirei*	Lichenised	100
101	Fungus	Uncl.	100
103	Fungus	Uncl.	100
104	*Vishniacozyma victoriae*	Yeast	100
105	*Mrakia frigida*	Yeast	100
106	Fungus	Uncl.	100
107	Fungus	Uncl.	100
108	Fungus	Uncl.	100
109	*Gelidatrema* sp.	Yeast	100
11	*Leucosporidium* sp.	Yeast	100
110	*Vishniacozyma* sp.	Yeast	100
111	Fungus	Uncl.	100
112	Microbotryomycete	Yeast	98
113	Fungus	Uncl.	100
114	Fungus	Uncl.	100
115	*Rhinocladiella* sp.	Yeast	100
116	*Polyblastia bryophila*	Lichenised	98
117	Ascomycete	Uncl.	88
118	Fungus	Uncl.	100
119	Fungus	Uncl.	100
12	*Vishniacozyma tephrensis*	Yeast	93
120	Fungus	Uncl.	100
121	Fungus	Uncl.	100
124	Ascomycete	Uncl.	87
125	Fungus	Uncl.	100
126	Ascomycete	Uncl.	100
127	Ascomycete	Uncl.	100

*Values derived from the RDP classifier against the UNITE database. Uncl., unclassifiable.

### Alpha diversity

**FIGURE 2:**
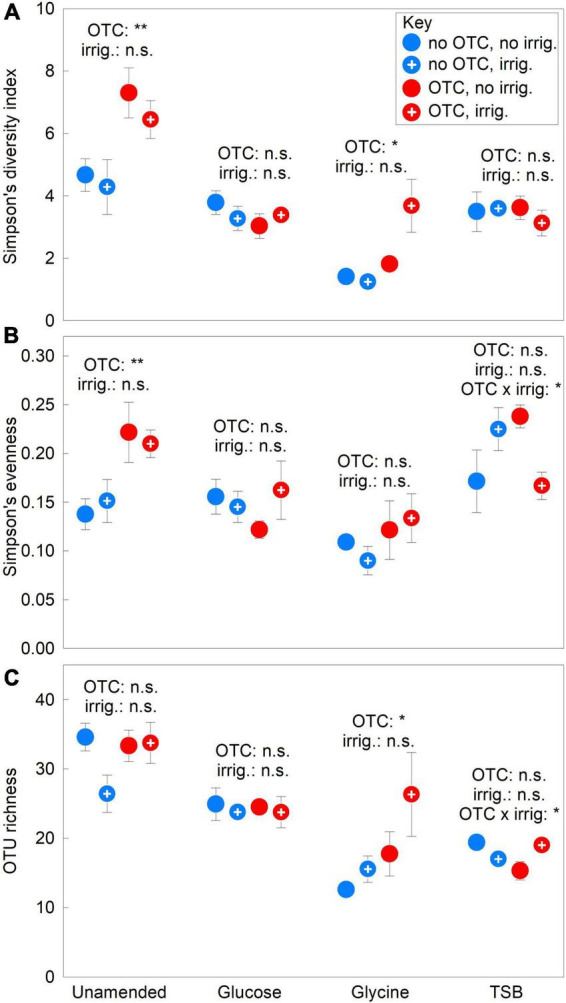
Treatment effects on soil fungal alpha diversity. **(A)** Simpson’s diversity index, **(B)** Simpson’s evenness and **(C)** OTU richness averaged over 2009–2012 in soils treated with a factorial combination of warming applied with open top chambers, irrigation and three organic substrates. Values are means of four replicates ± SEM. The main effects of OTCs and irrigation in unamended, glucose-, glycine- and TSB-amended soils are shown, along with significant OTC × irrigation interaction effects. OTC, open top chamber; irrig., irrigation; n.s., not significant.

The OTCs increased fungal community alpha diversity in the absence of substrates. In unamended soil, ANOVA showed that the chambers increased Simpson’s diversity index and evenness values by 49–54% in 2009–2012 (both *F*_1,12_ ≥ 10.29, *P* ≤ 0.008; Figures 2A,B) and by 102–142% in 2012 (both *F*_1,12_ ≥ 17.73, *P* ≤ 0.001; Supplementary Figures 3A, 4A). The chambers did not affect OTU richness in unamended soil (*F*_1,12_ = 1.08, *P* = 0.320; Figure 2C) and did not influence alpha diversity metrics in glucose-amended soils (all *F*_1,12_ ≤ 0.97, *P* ≥ 0.344; Figures 2A–C). In glycine-amended soils, Simpson’s diversity index values were approximately doubled, and OTU richness was 56% higher, in chambered compared with unchambered soil in 2009–2012 (both *F*_1,12_ = 4.92, *P* = 0.047; Figures 2A,C). However, the influence of OTCs on index values in soils treated with glycine diminished over the course of the experiment (Supplementary Figure 3C), with no effect being recorded in 2012 (*F*_1,12_ ≤ 3.60, *P* ≥ 0.079). Simpson’s diversity index and OTU richness values in glycine-amended soils were negatively associated in 2009–2012 with the relative abundance of *Pseudogymnoascus* sp., which accounted for 72–98% and 27–99% of the community in unchambered and chambered soils, respectively (Supplementary Figures 5A,B). In TSB-amended soils, although OTCs had no main effects on alpha diversity (all *F*_1,12_ ≤ 1.02, *P* ≥ 0.332), significant OTC × irrigation interactions were recorded on evenness and OTU richness in 2009–2012, with, in unchambered and chambered soils, irrigation causing increases and decreases in evenness, and decreases and increases in richness, respectively (both *F*_1,12_ ≥ 8.25, *P* ≤ 0.014; Figures 2B,C). Irrigation had no main effects on alpha diversity metrics in any soils in 2009–2012 (all *F*_1,12_ ≤ 2.58, *P* ≥ 0.134; Figures 2A–C).

The substrates reduced soil fungal alpha diversity. In 2009–2012, relative to unamended soils, glucose, glycine and TSB each decreased Simpson’s diversity index values and OTU richness by 18–63%, and glycine reduced evenness by 37% (all *F*_1,30_ ≥ 10.92, *P* ≤ 0.002; Figures 2A–C). Between 2009 and 2012, mean values of the Simpson’s index and evenness increased by 43–54% in unamended soils, and index values and OTU richness declined by 33–63% in soils receiving each substrate (Supplementary Figures 3, 4, 6).

### Beta diversity

**FIGURE 3:**
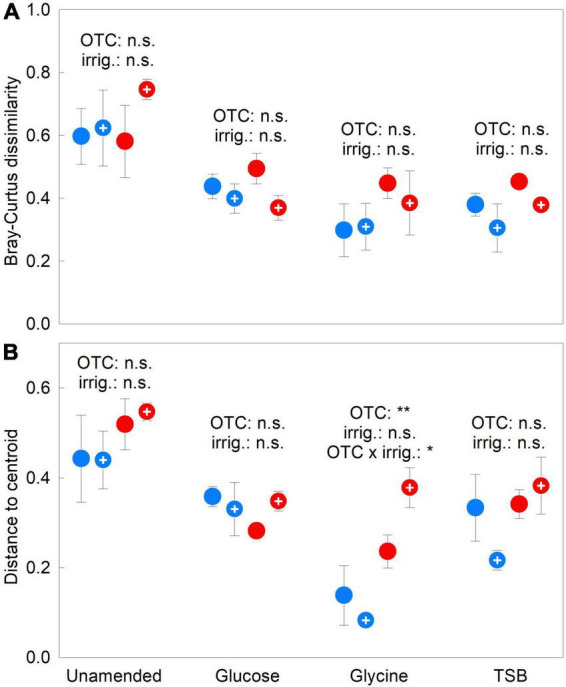
Treatment effects on soil fungal beta diversity. **(A)** Bray–Curtis dissimilarity and **(B)** distance to centroid values averaged over 2009–2012 in soils treated with a factorial combination of warming applied with open top chambers, irrigation and three organic substrates. Values are means of four replicates ± SEM. The main effects of OTCs and irrigation in unamended, glucose-, glycine- and TSB-amended soils are shown, along with a significant OTC × irrigation interaction effect. Notation and abbreviations as in Figure 2.

Bray–Curtis dissimilarity remained unaffected by OTCs, irrigation or the OTC × irrigation interaction in 2009–2012 (all *F*_1,12_ = 2.79, *P* ≥ 0.121; Figure 3A). Distance to centroid values were similarly unaffected by OTCs in unamended, glucose- and TSB-amended soils in 2009–2012, but, in glycine-amended soils, were more than doubled in chambered soils compared with unchambered soils (*F*_1,12_ = 19.66, *P* = 0.001; Figure 3B). The highest distance to centroid values were recorded in glycine-amended soils that were chambered and irrigated, accounting for the significant OTC × irrigation interaction (*F*_1,12_ = 4.97, *P* = 0.046; Figure 3B). As for Simpson’s index and OTU richness, distance to centroid values were negatively associated with the relative abundance of *Pseudogymnoascus* sp. (Supplementary Figure 5C). Compared with unamended soils, each of the substrates elicited 32–57% reductions in both beta diversity measures in 2009–2012 (all *F*_1,30_ ≥ 15.71, *P* ≤ 0.001; Figures 3A,B). Substrate effects on beta diversity became more pronounced over the duration of the experiment, with, between 2009 and 2012, 69–94% reductions in Bray-Curtis dissimilarity in all substrate-amended soils, and 44–46% decreases in distance to centroid values in soils receiving glucose and TSB (Supplementary Figures 7, 8).

### Community-level responses

PERMANOVA analysis indicated that the substrates were the predominant factor explaining variation in community composition, which they affected from 2009 onward. Although main effects of OTCs and irrigation were significant, their R^2^ values were at least an order of magnitude smaller than those for substrate amendment (Supplementary Figure 9). Variance partitioning showed that 36–64% of community composition variation was attributable to substrates at each sampling, with OTCs and irrigation accounting for <3% of variation (Supplementary Figure 9).

### Taxon-level responses to substrates

**FIGURE 4:**
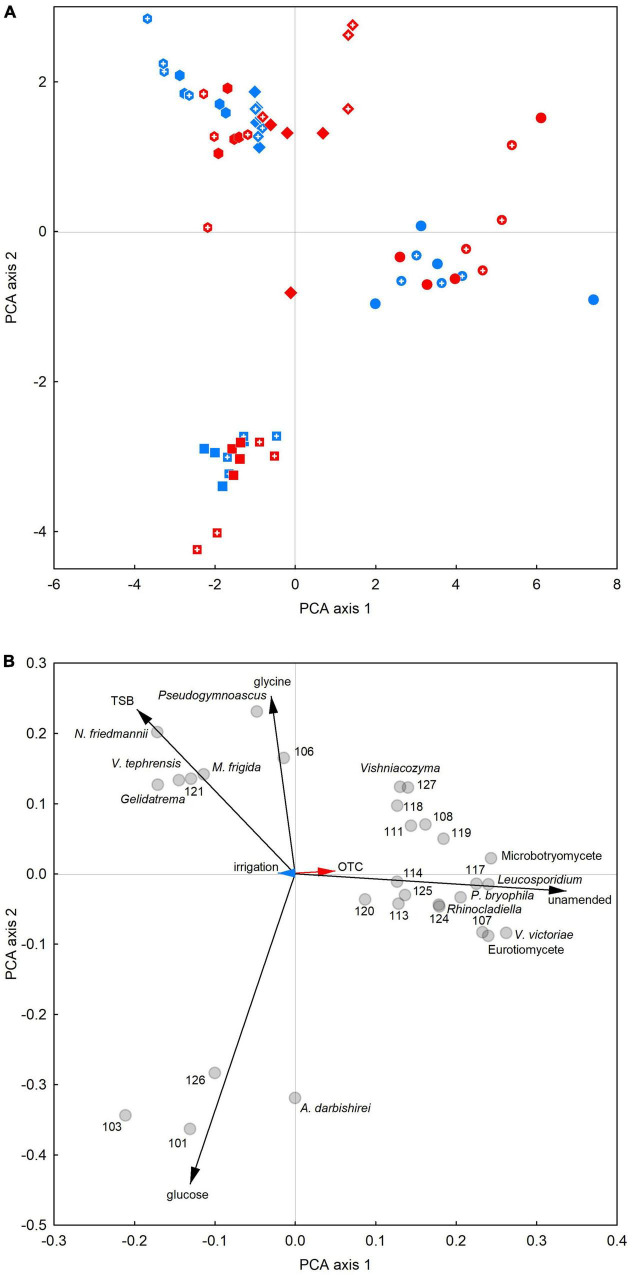
Principal components analysis of associations between the relative abundances of 30 frequent soil fungal taxa and treatments. **(A)** Score plot and **(B)** loading plot of data averaged over 2009–2012. Circles, squares, diamonds and hexagons in panel **(A)** denote soils to which no substrates, glucose, glycine or TSB were applied, respectively. Red, blue and black arrows in panel **(B)** denote OTC, irrigation and substrate vectors, respectively, and circles show the positions of each taxon. The numbers shown in panel **(B)** correspond to the OTUs named at kingdom or phylum level in Table 1.

Principal component analysis showed that unamended soils in 2009–2012 were dominated by a suite of fungi consisting of members of the Eurotiomycetes and Microbotryomycetes, *Vishniacozyma* sp., *V. victoriae* and *Leucosporidium* sp., *Rhinocladiella* sp., *P. bryophila* and 12 unclassifiable fungi (Figure 4). In glucose-amended soils, *A. darbishirei* and three unclassifiable fungi (OTUs 101, 103 and 126) dominated the soil fungal community (Figure 4). In contrast, PCA indicated that the relative abundance of *Pseudogymnoascus* sp. was positively associated with glycine amendment (Figure 4) and that the abundances of OTU 121 and the yeasts *N. friedmannii*, *M. frigida*, *V. tephrensis* and *Gelidatrema* sp. were positively associated with TSB application (Figure 4). PCA on data from individual samplings indicated similar responses to those recorded in 2009–2012 (Supplementary Figures 10A–D).

Factorial contrasts showed substrates to affect the abundances of 17 of the 30 most frequent OTUs in 2009–2012 (Table 2), with similar effects of the substrates being recorded at each sampling (Supplementary Table 1). In 2009–2012, the relative abundances of the Eurotiomycete, *V. victoriae, Leucosporidium* sp., two ascomycetes (OTUs 117 and 124) and two unclassifiable fungi (OTUs 107 and 111) were 3–18 fold higher in unamended soil than in glucose-, glycine- or TSB-amended soils (Table 2). Indicator species analyses similarly selected *Leucosporidium* sp., OTUs 117, 120, 124, *Rhinocladiella* sp. and 22 infrequent OTUs as taxa representative of unamended soils (Supplementary Table 2). In contrast, taxa positively associated with glucose amendment in the PCA (*A. darbishirei* and OTUs 101, 103 and 126) were each 2–100 fold higher in abundance in glucose-amended than in unamended soils (Table 2), with indicator species analysis selecting OTU 126 and eight infrequent OTUs as being representative of soils receiving glucose (Supplementary Table 2). As suggested by the PCA, factorial contrasts showed that, compared with unamended soils, the abundance of *Pseudogymnoascus* sp. increased by 64 fold in glycine-amended soils and the abundances of *N. friedmannii*, *M. frigida*, *Gelidatrema* sp., *V. tephrensis* and OTU 121 increased by 22–310 fold in TSB-amended soils (Table 2). Indicator species analyses showed *M. frigida*, *V. tephrensis*, OTU 121 and three infrequent OTUs to be representative of soils amended with TSB (Supplementary Table 2).

**TABLE 2:** Effects of substrate amendments on the relative abundances of 17 soil fungal OTUs in 2009–2012.

OTU number	Identity	Relative abundance (%)^†^			
		No substrate	Glucose	Glycine	TSB
1	Eurotiomycete	24.353 (16.915, 32.459)	5.128 (3.021, 7.436)***	1.549 (0.673, 2.528)***	1.837 (0.912, 3.042)***
104	*Vishniacozyma victoriae*	7.663 (4.975, 10.451)	1.505 (0.737, 2.540)*	0.503 (0.075, 1.077)***	0.555 (0.139, 1.190)***
107	Fungus	6.841 (4.722, 8.962)	0.908 (0.195, 2.348)**	0.171 (0.041, 0.336)***	0.451 (0.012, 1.374)***
11	*Leucosporidium* sp.	3.657 (2.064, 5.357)	0.090 (0.001, 0.213)***	0.379 (0.072, 0.850)**	0.096 (0.015, 0.207)***
111	Fungus	2.176 (1.049, 3.648)	0.070 (0.023, 0.136)***	0.892 (0.178, 2.122)	0.621 (0.097, 1.585)*

124	Ascomycete	1.775 (0.808, 3.051)	0.252 (0.121, 0.408)	0.162 (0.010, 0.396)**	0.232 (0.004, 0.668)**
117	Ascomycete	1.554 (0.671, 2.577)	0.275 (0.026, 0.623)	0.034 (0.006, 0.070)*	0.045 (0.002, 0.106)

101	Fungus	0.389 (0.107, 0.812)	35.415 (26.685, 43.793)*	0.514 (0.085, 1.391)	4.152 (1.252, 8.763)
100	*Austroplaca darbishirei*	8.542 (5.683, 12.590)	22.879 (14.704, 31.807)*	0.613 (0.229, 1.181)***	1.584 (0.745, 2.696)***
103	Fungus	0.201 (0.090, 0.332)	19.551 (16.527, 22.718)***	1.959 (1.095, 2.977)**	7.339 (5.444, 9.381)***
126	Ascomycete	0.029 (0.004, 0.059)	2.946 (1.357, 4.976)**	0.004 (0.001, 0.009)	0.187 (0.037, 0.390)

0	*Pseudogymnoascus* sp.	1.147 (0.460, 2.010)	0.351 (0.280, 0.420)	75.070 (63.700, 84.900)***	6.007 (2.805, 9.619)

10	*Naganishia friedmannii*	0.832 (0.356, 1.595)	0.857 (0.445, 1.379)	0.321 (0.148, 0.617)	41.335 (33.618, 49.057)***
105	*Mrakia frigida*	0.079 (0.033, 0.151)	0.055 (0.033, 0.087)	0.064 (0.045, 0.084)	15.863 (8.535, 24.522)***
109	*Gelidatrema* sp.	0.024 (0.006, 0.048)	1.427 (0.345, 2.786)	0.025 (0.013, 0.042)	7.471 (3.695, 11.764)***
12	*Vishniacozyma tephrensis*	0.138 (0.001, 0.420)	0.467 (0.157, 0.948)	0.004 (0.001, 0.007)	3.260 (1.595, 5.141)***
121	Fungus	0.029 (0.001, 0.073)	0.162 (0.060, 0.297)	0.004 (0.002, 0.005)	2.877 (1.037, 5.215)***

Note that OTUs are grouped and ordered by their relative abundances in unamended and glucose-, glycine- and TSB-amended soils, respectively. ^†^Values are means of 16 replicates averaged over 2009–2012, with lower and upper 95% bootstrap confidence intervals in parentheses. Asterisks denote significant differences from the no substrate mean at *P < 0.05, **P < 0.01, and ***P < 0.001, following Bonferroni correction.

### Taxon-level responses to open top chambers and irrigation

**FIGURE 5:**
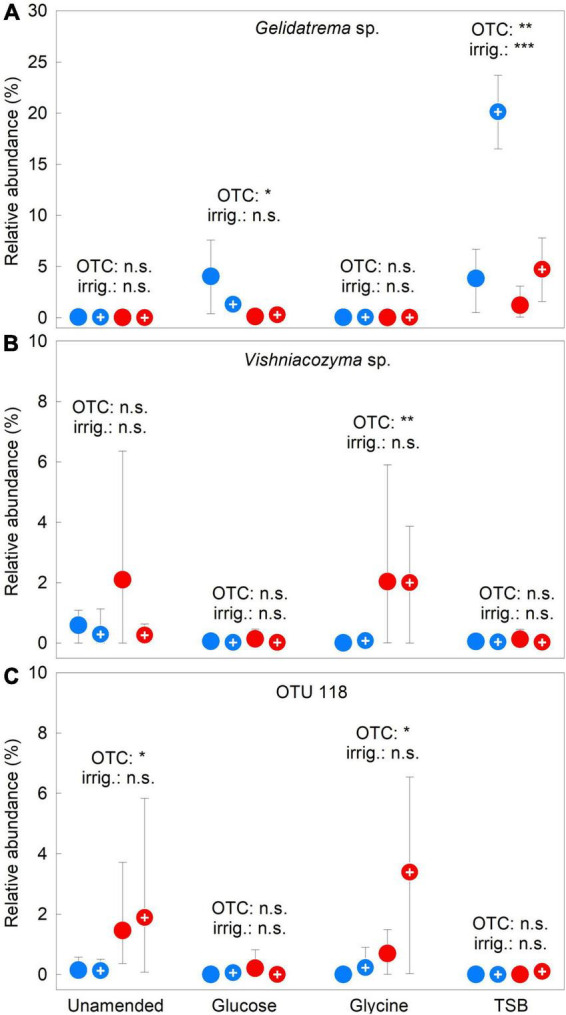
Treatment effects on the relative abundances of three soil fungal taxa. Mean relative abundances of **(A)** *Gelidatrema* sp., **(B)** *Vishniacozyma* sp., and **(C)** OTU 118 averaged over 2009–2012 in soils treated with a factorial combination of warming applied with open top chambers, irrigation and three organic substrates. Values are means of four replicates ± 95% bootstrap confidence intervals. Notation and abbreviations as in Figure 2. Note that *y*-axes are not identically scaled.

In agreement with PERMANOVA analyses, PCA showed OTCs and irrigation to have less pronounced effects on the abundances of frequent OTUs than the substrates, with shorter vectors for these treatments than those for glucose, glycine or TSB (Figure 4). Following Bonferroni correction, factorial contrasts showed that *Gelidatrema* sp. responded to OTCs and irrigation, with, in 2009–2012, chambers reducing its abundance by 78%, compared with unchambered soil (*F*_1,48_ = 25.72, *P* = 0.000006) and irrigation increasing its abundance by 1.9 fold, relative to unirrigated soil (*F*_1,48_ = 11.11, *P* = 0.0016; Figure 5A). Indicator species analyses showed *Vishniacozyma* sp. and OTUs 118 and 127 to be representative of chambered soils (Supplementary Table 3). Analyses using factorial contrasts similarly indicated that OTCs increased the abundances of *Vishniacozyma* sp. and OTU 118 in glycine-amended soils by 45- and 12-fold, respectively, and increased the abundance of OTU 118 in unamended soil by 11 fold (all *F*_1,14_ ≥ 7.00, *P* ≤ 0.019; Figures 5B,C). Indicator species analyses also identified eight and two infrequent OTUs as being representative of chambered and unchambered soils, respectively (Supplementary Table 3). No effects of irrigation on individual OTUs were apparent from the PCA (Figure 4), with factorial contrasts and indicator species analyses similarly failing to identify taxa significantly associated with this treatment.

### DNA concentrations

**FIGURE 6:**
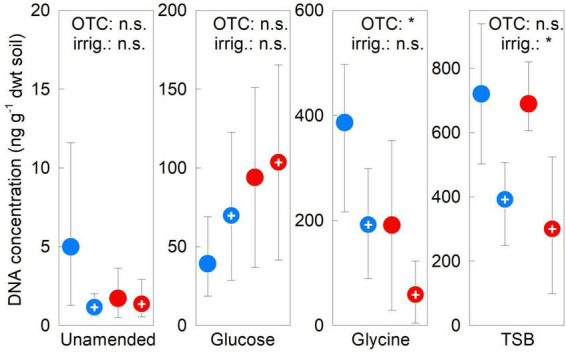
Treatment effects on soil fungal DNA concentrations. Mean fungal DNA concentrations averaged over 2009–2012 in soils treated with a factorial combination of warming applied with open top chambers, irrigation and three organic substrates. Values are means of four replicates ± 95% bootstrap confidence intervals. Notation and abbreviations as in Figure 2. Note that *y*-axes are not identically scaled.

Factorial contrasts showed no effects of OTCs or irrigation on fungal DNA concentrations in unamended or glucose-amended soils in 2009–2012 (all *F*_1,12_ ≤ 1.50, *P* ≥ 0.244; Figure 6). In contrast, these analyses showed OTCs in 2009–2012 to reduce DNA concentration by 57% in glycine-amended soils (*F*_1,12_ = 6.11, *P* = 0.029), and irrigation in TSB-amended soils to halve DNA concentration (*F*_1,12_ = 9.04, *P* = 0.011; Figure 6). The OTC × irrigation interaction did not affect DNA concentrations in any soils in 2009–2012 (all *F*_1,12_ ≤ 1.91, *P* ≥ 0.193). DNA concentrations did not alter in unamended soils between 2009 and 2012, but increased by 1–2 orders of magnitude in substrate-amended soils over this period (Supplementary Figure 11).

## Discussion

Experimental warming of vegetated soils typically increases fungal community size (Yergeau et al., 2012; Kim et al., 2018; Salazar et al., 2020), but, in contrast, exerts only weak effects on fungal community diversity (Jumpponen and Jones, 2014; Geml et al., 2015, 2021; Semenova et al., 2015; Van Nuland et al., 2020; Pec et al., 2021). Here, we tested the effects of warming applied with OTCs on a maritime Antarctic fellfield soil fungal community that consisted of yeasts, filamentous and lichenised fungi (Atlas et al., 1978; Newsham et al., 2021) and was conspicuous by its lack of mycorrhizal symbionts (Newsham et al., 2009). Strikingly, contrary to our expectations and findings from similar studies in maritime Antarctica (Yergeau et al., 2012; Kim et al., 2018), OTCs more than doubled fungal community alpha diversity in unamended soil after 5 years. Other than positive effects of OTCs on diversity in glycine-amended soils, which were attributable to an abundance of *Pseudogymnoascus*, no other main effects of chambers were recorded on community diversity in soils treated with substrates. These, and other findings, which in part help to explain the weak effects of warming on the diversity of fungal communities in vegetated soils, are discussed below.

### Experimental warming increases fungal alpha diversity in unamended fellfield soil

Surface air temperature and liquid water availability are strongly associated with each other in Antarctic habitats (Turner et al., 1997), and it was hence unclear from a space-for-time substitution study which of these two factors causes increased fungal OTU richness in more northerly maritime Antarctic soils (Newsham et al., 2016). In the present study, although chambers did not influence OTU richness in unamended soils, their positive effects on Simpson’s diversity index and evenness in these soils suggests that the 1.7°C rise in soil temperature that they elicited during midsummer (Misiak et al., 2021) increased the abundances of infrequent taxa in the community, and, as shown by the indicator species analyses, led to higher frequencies in soil of *Vishniacozyma* and unclassifiable fungi. The precise reasons for the higher abundances of these fungal taxa in warmed soils are obscure, but may be owing to increased thermal input enhancing their metabolic activities and causing a switch from survival to growth and dispersal strategies (Green et al., 2011). Whilst we cannot exclude the possibility that substantial rises in precipitation in maritime Antarctica will influence soil fungal diversity, the higher Simpson’s diversity index and evenness values recorded in chambered and unamended soils, with no apparent effects of irrigation, suggest that increased temperature alone elicits higher alpha diversity of maritime Antarctic fellfield soil fungal communities. Although the analyses here were based on DNA in both active and inactive cells, rather than RNA in live, actively transcribing cells (Cox et al., 2019), our observation of increased alpha diversity in warmed, unamended soil is unlikely to have arisen from stochastic differences in inactive or dead cells between chambered and unchambered soils, but instead from changes to the dynamics of the active fungal community. Our observation is broadly consistent with regional and continental-scale studies across climatic gradients showing that mean annual temperature correlates positively with ectomycorrhizal fungal diversity in North American eastern temperate forests (Steidinger et al., 2020) and that temperature is a better correlate for forest soil fungal richness than edaphic factors (Zhou et al., 2016). At the global scale, however, although temperature is the best predictor for the distribution of 457 frequent soil fungal taxa (Větrovský et al., 2019), its relationship with soil fungal community alpha diversity appears to dissipate, with studies reporting both declines and increases in species richness at greater distances from the equator (Tedersoo et al., 2014; Větrovský et al., 2019).

### Responses of fungal guilds to warming

In the absence of higher animals and plants from the soil studied at Mars Oasis, it is reasonable to assume that, as for other maritime Antarctic fellfield soils (Newsham et al., 2021), the yeasts and filamentous fungi inhabiting it function predominantly as saprotrophs. The abundance of this guild in soil at Mars Oasis may help to explain the observed increase in community alpha diversity in warmed and unamended soil, since previous studies have shown that the species richness of saprotrophic fungi responds positively to warming in High- and Low Arctic soils and temperate forest soil (Geml et al., 2015; Mundra et al., 2016; Pec et al., 2021). In contrast with the Arctic, where the richness of the lichenised fungal guild declines in warmer habitats (Lang et al., 2012; Geml et al., 2015) – which is possibly owing to competition from plants (Timling et al., 2014; Newsham et al., 2021) – the diversity of lichens and lichen-forming soil fungi in maritime Antarctica is positively associated with temperature, with higher species richness at lower latitudes (Peat et al., 2007; Green et al., 2011) being best predicted by surface air temperature (Newsham et al., 2021). It is thus possible that increased diversity of lichenised fungi, and notably members of the Verrucariaceae (Newsham et al., 2016), may also in part explain the rise in community alpha diversity in warmed and unamended soil at Mars Oasis. An additional factor that may have led to increased community alpha diversity is the absence of ectomycorrhizal fungi from this soil. Studies in mesic Low Arctic soils have repeatedly shown that the diversity of this guild, which is abundant under woody plant species, is strongly diminished by warming treatments (Geml et al., 2015, 2021; Morgado et al., 2015). This raises the possibility that the neutral effects of warming on total fungal community diversity observed under woody plants after 3–20 years of treatment (Semenova et al., 2015; Van Nuland et al., 2020; Pec et al., 2021) may arise from reductions in ectomycorrhizal fungal alpha diversity masking the positive effects of warming on other guilds.

### Effects of organic C and N inputs on fungal alpha diversity

The weak effect of warming on fungal community alpha diversity identified by studies at lower latitudes might also arise from reductions in diversity caused by organic C and N inputs. A common feature of these studies is that they have been conducted in vegetated soils with organic C and N concentrations of 1–7% and 0.1–0.5%, respectively (Kim et al., 2018; Van Nuland et al., 2020; Geml et al., 2021). As shown here, the amendment of oligotrophic soil with organic substrates that raised mean soil organic C concentrations from 0.26 to 0.36–0.39% and N concentrations from 0.02 to 0.05–0.08% diminished soil fungal alpha diversity by 18–63%. Studies in High Arctic soils similarly indicate that elevated concentrations of organic C and N associated with plant cover suppress fungal diversity and reduce the number of unique fungal taxa (Grau et al., 2017; Canini et al., 2019). It is hence plausible that elevated concentrations of organic C and N associated with litter inputs and rhizodeposition in vegetated soils (Jones et al., 2004) diminish fungal community alpha diversity and hence attenuate the response of the community to warming.

### Effects of warming on fungal diversity in substrate-amended soils

Open top chambers had no effects on diversity in glucose-amended soils, and similarly did not affect diversity in soils amended with TSB, with interaction effects of OTCs and irrigation on evenness and richness instead being found in the latter soils. The only main effects of OTCs on diversity were recorded in glycine-amended soils, with higher Simpson’s diversity index, OTU richness and distance to centroid values being recorded in chambered than in unchambered soils receiving the amino acid. As in the High Arctic, where the amendment of permafrost soils with plant litter causes substantial increases in the abundance of *Pseudogymnoascus* (Adamczyk et al., 2020), an OTU of this genus accounted for ≥ 72% of the fungal community in unchambered, glycine-amended soils. However, in agreement with the strong inhibitory effect of warming on the abundance of *Pseudogymnoascus* (Misiak et al., 2021), the OTU accounted for as little as 27% of the community in chambered soils amended with glycine. Given the strong negative associations recorded here between the abundance of this taxon and diversity, we interpret the higher alpha and beta diversity recorded in chambered, glycine-amended soils as having arisen from decreased competitive exclusion of other taxa by this rapidly growing filamentous fungus (Misiak et al., 2021).

### Substrates rapidly alter soil fungal community composition and diminish beta diversity

Substrates were identified as the main factor explaining variation in community composition, with measurable effects on the community occurring in substrate-amended soils within 2 years of treatment. Reductions in Bray-Curtis dissimilarity and distance to centroid values were recorded in these soils, indicative of an overall homogenization in community composition, with turnover rates rapidly declining and then stabilizing following amendment. The rapidity of these changes in soils that are thawed for only 12–14 weeks each year (Misiak et al., 2021) suggests shifts in fungal community composition, and possibly activity (Mikan et al., 2002; McMahon et al., 2009), at sub-zero temperatures. Organic N inputs to temperate forest soils similarly suppress fungal richness and decrease variation between communities after just 4 months (Cline et al., 2018). Furthermore, in sparsely vegetated High Arctic permafrost soils, litter inputs reduce fungal beta diversity, increase community homogeneity, and, as observed here in TSB-amended soils, increase the abundances of the yeasts *Vishniacozyma*, *Mrakia* and *Naganishia* after 1 year (Adamczyk et al., 2020). Much slower response times have been recorded in densely vegetated Low Arctic soils, with 15–18 year lag times in the responses of ectomycorrhizal root tip communities and soil fungal biomass to fertilisation (Rinnan et al., 2007, 2013; Deslippe et al., 2011). At present, the reasons for the slower responses of Low Arctic soil fungal communities to fertilisation remain obscure. However, it appears that competition from roots for organic C and N compounds, including glycine (Lipson and Monson, 1998), may partly account for the delayed fungal community responses in these soils, with the gradual shifts in Arctic soil microbial communities instead arising from changes to belowground plant biomass (Rinnan et al., 2007).

### Yeasts and their responses to warming and irrigation

A previous study has shown the yeast genera *Candida* and *Trichosporon* to be over-represented in metabarcoding analyses using the internal transcribed spacer 1 region as a marker (Hoggard et al., 2018). However, neither of these animal pathogens was recorded in the soil at Mars Oasis (Davey and Newsham, 2021), most probably indicating their absence from soil and suggesting a minimal influence of marker choice on the inferred taxonomic composition of the yeast component of the fungal community. Irrigation and OTCs were found to increase and decrease the abundance of *Gelidatrema* sp. by 1.9-fold and 78%, respectively. The positive effect of irrigation on this yeast most probably arose from its accelerated growth at higher water availability, whereas the negative effect of OTCs might be explained by the temperatures of chambered soil at Mars Oasis exceeding 20°C for up to 38 h each month in midsummer (Misiak et al., 2021). A related species, *G. psychrophila*, which inhabits High Arctic ice islands, cannot grow at 25°C (Tsuji et al., 2018), and it is thus plausible that, as for *Pseudogymnoascus* (Misiak et al., 2021), temperatures of >20°C inhibit the growth of *Gelidatrema* in Mars Oasis soil. Indicator species analysis showed another yeast, a *Vishniacozyma* sp., along with two unassignable taxa, to be representative of chambered soils, with the yeast increasing in abundance by 45 fold in warmed, glycine-amended soils. This was an unexpected finding, since, although present in temperate soils (Mašínová et al., 2017), members of *Vishniacozyma* are often encountered in polar environments, such as periglacial High Arctic habitats (Perini et al., 2019), in which, like *G. psychrophila*, they exhibit strong psychrotrophy (Tsuji et al., 2019).

### Responses of fungal DNA concentrations to warming and substrates

Contrary to our expectations and observations from previous studies (Yergeau et al., 2012; Kim et al., 2018), we found no evidence to support the view that warming increases fungal DNA concentrations in maritime Antarctic fellfield soil. We postulate that the development of fungal biomass in chambered soils at Mars Oasis would have been constrained by their aridity, and, in unamended soils, by the 73–99% lower concentrations of C and N measured in them than in previously studied soils (Yergeau et al., 2012; Kim et al., 2018). In contrast, and as expected, warming in combination with substrates altered soil fungal DNA concentrations, with OTCs reducing fungal DNA concentrations by 57% in glycine-amended soil. Previous studies have attributed similar reductions in fungal biomass in chambered soils to the desiccating effects of OTCs (Allison and Treseder, 2008; Christiansen et al., 2017). However, in the absence of effects of OTCs at Mars Oasis on soil water potential, reductions in the abundance of *Pseudogymnoascus* in chambered, glycine-amended soils (Misiak et al., 2021) most probably explain the negative influence of warming on DNA concentrations in soils treated with the amino acid. Similarly, the reductions in the abundance of this genus in irrigated soils receiving TSB (Misiak et al., 2021) most probably also account for the negative effects of irrigation on DNA concentrations in TSB-amended soils recorded here.

### Fungal alpha diversity in maritime Antarctic soils during the 21st century

The observations here indicate that the alpha diversity of fungal communities inhabiting the oligotrophic fellfield soils that are widespread across maritime Antarctica will rise as they warm over future decades (Bracegirdle et al., 2020). However, they also suggest that the accumulation of organic C and N compounds in fellfield soils arising from expanding plant populations (Yergeau et al., 2007; Strauss et al., 2009; Grau et al., 2017) are likely, in time, to diminish the positive effects of warming on fungal alpha diversity. Given the pivotal role of soil fungi in terrestrial ecosystem productivity (Swift et al., 1979), we advocate studies to clarify whether increased diversity of maritime Antarctic soil fungal communities – and particularly that of their saprotrophic members – will accelerate C and N cycling (Setälä and McLean, 2004), and might hence be instrumental in the plant community expansions that are anticipated across the region during the present century (Fowbert and Smith, 1994; Royles et al., 2013; Cannone et al., 2022).

## Conclusion

A 1.7°C rise in the mean summertime temperature of an oligotrophic fellfield soil was found to increase Simpson’s diversity index and evenness values by 102–142% in a 5 year field experiment at Mars Oasis in the southern maritime Antarctic. These positive effects of warming on soil fungal community alpha diversity, which support previous data from a space-for-time substitution study (Newsham et al., 2016), are attributable to an abundance of saprotrophic and lichenised fungi in the soil at Mars Oasis. Increased concentrations of organic C and N reduced fungal community alpha diversity by 18–63%, suggesting that rhizodeposition and litter inputs from plants as they colonise newly exposed fellfield soils (Jones et al., 2004; Strauss et al., 2009; Lee et al., 2017) will attenuate the positive effects of increased temperature on maritime Antarctic soil fungal diversity as the region warms during the 21st century (Bracegirdle et al., 2020).

## Data availability statement

The datasets generated for this study can be found in the NCBI Sequence Read Archive (accession code PRJNA798896) and in Davey and Newsham (2021). Further results can be found in the Supplementary material.

## Author contributions

KN, DH, and LB conceived the study. KN conducted the fieldwork. WG-C and MSD conducted the labwork along with MM. MLD and KN analyzed the data and wrote the manuscript. All authors approved the submitted version.
