# Supplementary material for: Experimental warming increases fungal alpha diversity in an oligotrophic maritime Antarctic soil
Source: Front Microbiol. 2022 Nov 10;13:1050372. doi: 10.3389/fmicb.2022.1050372 (PMC9684652; doi:10.3389/fmicb.2022.1050372)
Supplement: Supplementary file 1 [file Data_Sheet_1.docx]

**Supplementary Table 1 |** Effects of substrates on relative abundances of 17 fungal taxa at each sampling between 2009 and 2012. Note that taxa are grouped by their relative abundances in unamended and glucose-, glycine- and TSB-amended soils, respectively, and that only taxa which responded to the treatments in each year are shown.

| Year |  | OTU number |  | Identity |  | Relative abundance (%)^†^ | | | |
| --- | --- | --- | --- | --- | --- | --- | --- | --- | --- |
|  |  |  |  |  |  | Unamended | Glucose | Glycine | TSB |
| 2009 |  | 1 |  | Eurotiomycete |  | 32.162 (20.271, 44.134) | 14.022 (7.771, 20.703)* | 4.634 (1.489, 8.372)*** | 6.637 (3.104, 11.732)*** |
|  |  | 104 |  | *Vishniacozyma victoriae* |  | 9.353 (4.462, 15.421) | 3.094 (0.922, 6.616) | 1.247 (0.192, 2.854)** | 2.163 (0.467, 4.696)* |
|  |  | 107 |  | Fungus |  | 8.434 (2.581, 14.058) | 1.193 (0.150, 3.021)* | 0.021 (0.002, 0.057)** | 1.788 (0.033, 5.715)* |
|  |  |  |  |  |  |  |  |  |  |
|  |  | 100 |  | *Austroplaca darbishirei* |  | 8.353 (4.525, 12.419) | 28.821 (18.633, 39.126)*** | 1.249 (0.449, 2.253) | 5.017 (2.099, 8.257) |
|  |  | 101 |  | Fungus |  | 0.099 (0.032, 0.223) | 17.288 (9.698, 25.639)*** | 1.645 (0.027, 5.675) | 7.571 (2.456, 15.774) |
|  |  | 103 |  | Fungus |  | 0.032 (0.001, 0.091) | 15.643 (10.696, 21.375)* | 2.238 (0.599, 4.370) | 10.871 (7.192, 15.030) |
|  |  | 126 |  | Ascomycete |  | 0.050 (0.000, 0.128) | 1.399 (0.589, 2.363)* | 0.000 (0.000, 0.000) | 0.615 (0.032, 1.335) |
|  |  |  |  |  |  |  |  |  |  |
|  |  | 0 |  | *Pseudogymnoascus* sp. |  | 0.721 (0.169, 1.411) | 0.201 (0.144, 0.264) | 66.460 (48.410, 82.471)*** | 5.945 (1.465, 11.582) |
|  |  |  |  |  |  |  |  |  |  |
|  |  | 10 |  | *Naganishia friedmannii* |  | 0.377 (0.080, 0.867) | 1.642 (0.502, 3.421) | 0.175 (0.070, 0.386) | 26.477 (17.139, 36.275)*** |
|  |  | 109 |  | *Gelidatrema* sp. |  | 0.027 (0.002, 0.075) | 1.676 (0.266, 3.753) | 0.031 (0.010, 0.059) | 5.915 (3.058, 8.868)*** |
|  |  | 12 |  | *Vishniacozyma tephrensis* |  | 0.453 (0.000, 1.526) | 0.480 (0.085, 1.284) | 0.006 (0.001, 0.014) | 4.992 (1.397, 9.319)* |
|  |  | 121 |  | Fungus |  | 0.001 (0.000, 0.004) | 0.360 (0.032, 0.817) | 0.002 (0.000, 0.005) | 2.870 (0.757, 5.974)* |
|  |  | 105 |  | *Mrakia frigida* |  | 0.010 (0.003, 0.020) | 0.011 (0.001, 0.023) | 0.010 (0.003, 0.018) | 2.862 (0.349, 5.766)* |
|  |  |  |  |  |  |  |  |  |  |
| 2010 |  | 1 |  | Eurotiomycete |  | 4.886 (0.042, 16.943) | 3.397 (1.094, 6.537) | 0.572 (0.165, 1.241)* | 0.408 (0.181, 0.725)* |
|  |  | 11 |  | *Leucosporidium* sp. |  | 6.601 (0.000, 16.24) | 0.302 (0.000, 0.872)* | 0.223 (0.000, 0.753)** | 0.007 (0.001, 0.019)** |
|  |  |  |  |  |  |  |  |  |  |
|  |  | 100 |  | *Austroplaca darbishirei* |  | 4.938 (0.065, 17.016) | 30.622 (16.525, 44.598)*** | 0.566 (0.179, 1.235) | 0.691 (0.333, 1.078) |
|  |  | 101 |  | Fungus |  | 0.239 (0.020, 0.716) | 25.779 (15.469, 36.074)*** | 0.143 (0.042, 0.312) | 6.193 (1.222, 12.971) |
|  |  | 103 |  | Fungus |  | 0.000 (0.000, 0.000) | 20.917 (13.557, 28.387)*** | 1.956 (0.929, 3.193) | 8.420 (5.678, 11.494)* |
|  |  | 126 |  | Ascomycete |  | 0.024 (0.000, 0.086) | 4.683 (1.018, 8.844)* | 0.011 (0.000, 0.030) | 0.118 (0.010, 0.268) |
|  |  |  |  |  |  |  |  |  |  |
|  |  | 0 |  | *Pseudogymnoascus* sp. |  | 0.248 (0.149, 0.345) | 0.517 (0.320, 0.927) | 84.004 (68.640, 94.929)*** | 4.559 (2.121, 7.248) |
|  |  |  |  |  |  |  |  |  |  |
|  |  | 10 |  | *Naganishia friedmannii* |  | 0.316 (0.126, 0.698) | 0.990 (0.340, 1.784) | 0.146 (0.122, 0.172) | 47.606 (39.645, 56.116)*** |
|  |  | 105 |  | *Mrakia frigida* |  | 0.094 (0.010, 0.288) | 0.038 (0.023, 0.054) | 0.068 (0.037, 0.103) | 14.117 (5.398, 23.700)* |
|  |  | 109 |  | *Gelidatrema* sp. |  | 0.020 (0.003, 0.038) | 1.333 (0.046, 3.122) | 0.027 (0.009, 0.058) | 9.407 (3.800, 15.683)* |
|  |  | 12 |  | *Vishniacozyma tephrensis* |  | 0.005 (0.000, 0.019) | 0.827 (0.095, 2.267) | 0.006 (0.001, 0.015) | 2.965 (1.341, 4.995)* |
|  |  | 121 |  | Fungus |  | 0.003 (0.000, 0.010) | 0.115 (0.005, 0.278) | 0.004 (0.001, 0.008) | 2.476 (0.573, 4.638)* |
|  |  |  |  |  |  |  |  |  |  |
| 2011 |  | 1 |  | Eurotiomycete |  | 24.677 (15.673, 35.766) | 1.256 (0.311, 2.389)*** | 0.264 (0.078, 0.552)*** | 0.214 (0.081, 0.459)*** |
|  |  | 107 |  | Fungus |  | 8.721 (3.852, 13.864) | 1.493 (0.070, 4.436)** | 0.170 (0.012, 0.398)*** | 0.004 (0.00, 0.008)*** |
|  |  | 11 |  | *Leucosporidium* sp. |  | 4.335 (1.879, 7.195) | 0.000 (0.000, 0.000)*** | 0.000 (0.000, 0.000)*** | 0.000 (0.000, 0.000)*** |
|  |  | 104 |  | *Vishniacozyma victoriae* |  | 4.047 (1.303, 7.769) | 0.751 (0.291, 1.444)* | 0.018 (0.00, 0.045)** | 0.004 (0.00, 0.010)** |
|  |  | 124 |  | Ascomycete |  | 2.594 (0.830, 4.860) | 0.049 (0.000, 0.172)* | 0.010 (0.000, 0.029)* | 0.001 (0.000, 0.002)** |
|  |  | 111 |  | Fungus |  | 2.448 (0.700, 4.742) | 0.021 (0.000, 0.055)* | 0.311 (0.010, 0.745)* | 0.004 (0.000, 0.012)* |
|  |  | 117 |  | Ascomycete |  | 1.223 (0.302, 2.314) | 0.000 (0.000, 0.000)* | 0.041 (0.000, 0.127)* | 0.002 (0.000, 0.007)** |
|  |  |  |  |  |  |  |  |  |  |
|  |  | 101 |  | Fungus |  | 0.332 (0.120, 0.681) | 45.931 (34.565, 56.451)*** | 0.122 (0.076, 0.179) | 2.142 (0.286, 5.702) |
|  |  | 103 |  | Fungus |  | 0.192 (0.074, 0.358) | 21.865 (18.362, 25.376)*** | 1.984 (0.811, 3.202) | 6.863 (4.942, 8.967)*** |
|  |  | 100 |  | *Austroplaca darbishirei* |  | 8.794 (4.128, 16.125) | 18.799 (10.410, 29.228)* | 0.516 (0.059, 1.358) | 0.522 (0.082, 1.530) |
|  |  | 126 |  | Ascomycete |  | 0.035 (0.000, 0.095) | 2.493 (1.023, 4.143)*** | 0.006 (0.000, 0.021) | 0.004 (0.000, 0.012) |
|  |  |  |  |  |  |  |  |  |  |
|  |  | 0 |  | *Pseudogymnoascus* sp. |  | 1.148 (0.372, 2.259) | 0.364 (0.269, 0.481) | 73.439 (60.150, 86.248)*** | 4.511 (1.282, 9.006) |
|  |  |  |  |  |  |  |  |  |  |
|  |  | 10 |  | *Naganishia friedmannii* |  | 1.902 (0.447, 3.893) | 0.347 (0.206, 0.513) | 0.715 (0.166, 1.614) | 48.838 (39.227, 57.922)*** |
|  |  | 105 |  | *Mrakia frigida* |  | 0.136 (0.030, 0.378) | 0.046 (0.014, 0.088) | 0.098 (0.057, 0.149) | 20.628 (10.206, 33.097)*** |
|  |  | 109 |  | *Gelidatrema* sp. |  | 0.005 (0.001, 0.008) | 1.611 (0.288, 3.898) | 0.026 (0.008, 0.055) | 6.791 (2.423, 12.334)** |
|  |  | 12 |  | *Vishniacozyma tephrensis* |  | 0.081 (0.000, 0.200) | 0.285 (0.114, 0.480) | 0.001 (0.000, 0.004) | 3.344 (1.635, 5.348)*** |
|  |  | 121 |  | Fungus |  | 0.000 (0.000, 0.000) | 0.090 (0.021, 0.169) | 0.006 (0.000, 0.013) | 2.173 (0.739, 3.972)** |
|  |  |  |  |  |  |  |  |  |  |
| 2012 |  | 1 |  | Eurotiomycete |  | 22.868 (11.954, 36.578) | 1.653 (0.608, 3.095)*** | 0.502 (0.085, 1.112)*** | 0.105 (0.068, 0.132)*** |
|  |  | 104 |  | *Vishniacozyma victoriae* |  | 12.433 (5.061, 20.552) | 0.297 (0.116, 0.529)*** | 0.007 (0.001, 0.016)*** | 0.019 (0.003, 0.038)*** |
|  |  | 107 |  | Fungus |  | 5.847 (1.821, 10.616) | 0.190 (0.020, 0.433)** | 0.007 (0.002, 0.013)** | 0.017 (0.000, 0.005)** |
|  |  | 111 |  | Fungus |  | 4.493 (1.481, 8.435) | 0.007 (0.002, 0.013)** | 0.223 (0.006, 0.577)** | 0.006 (0.000, 0.019)** |
|  |  | 11 |  | *Leucosporidium* sp. |  | 3.237 (0.465, 6.528) | 0.004 (0.000, 0.012)* | 0.001 (0.000, 0.005)* | 0.000 (0.000, 0.000)* |
|  |  | 124 |  | Ascomycete |  | 1.795 (0.650, 3.150) | 0.099 (0.000, 0.282)** | 0.000 (0.000, 0.000)** | 0.001 (0.000, 0.003)** |
|  |  | 117 |  | Ascomycete |  | 0.518 (0.127, 0.973) | 0.001 (0.000, 0.003)* | 0.022 (0.000, 0.068)* | 0.000 (0.000, 0.000)* |
|  |  |  |  |  |  |  |  |  |  |
|  |  | 101 |  | Fungus |  | 0.696 (0.062, 1.718) | 45.571 (35.463, 56.921)*** | 0.135 (0.041, 0.264) | 0.703 (0.111, 1.808) |
|  |  | 103 |  | Fungus |  | 0.412 (0.113, 0.748) | 21.308 (14.768, 27.523)*** | 1.834 (0.593, 3.402) | 3.201 (1.588, 5.176) |
|  |  | 100 |  | *Austroplaca darbishirei* |  | 10.458 (7.010, 13.890) | 17.878 (9.442, 26.877) | 0.139 (0.039, 0.345)* | 0.107 (0.069, 0.153)* |
|  |  | 126 |  | Ascomycete |  | 0.000 (0.000, 0.000) | 4.023 (1.815, 6.664)*** | 0.000 (0.000, 0.000) | 0.010 (0.000, 0.032) |
|  |  |  |  |  |  |  |  |  |  |
|  |  | 0 |  | *Pseudogymnoascus* sp. |  | 1.648 (0.283, 3.537) | 0.380 (0.283, 0.495) | 76.916 (66.715, 87.369)*** | 9.011 (1.811, 17.328) |
|  |  |  |  |  |  |  |  |  |  |
|  |  | 10 |  | *Naganishia friedmannii* |  | 0.392 (0.153, 0.719) | 0.450 (0.252, 0.782) | 0.159 (0.113, 0.217) | 42.418 (31.601, 53.363)*** |
|  |  | 105 |  | *Mrakia frigida* |  | 0.071 (0.048, 0.096) | 0.113 (0.058, 0.205) | 0.087 (0.048, 0.134) | 25.844 (14.171, 38.590)*** |
|  |  | 109 |  | *Gelidatrema* sp. |  | 0.044 (0.004, 0.125) | 1.309 (0.289, 2.673) | 0.014 (0.004, 0.031) | 7.771 (2.595, 13.391)** |
|  |  | 121 |  | Fungus |  | 0.099 (0.001, 0.244) | 0.081 (0.031, 0.149) | 0.002 (0.000, 0.004) | 3.990 (1.448, 7.338)** |
|  |  | 12 |  | *Vishniacozyma tephrensis* |  | 0.000 (0.000, 0.000) | 0.464 (0.185, 0.773) | 0.001 (0.000, 0.002) | 1.738 (0.909, 2.905)*** |

^†^Values are means of 16 replicates with lower and upper 95% bootstrap confidence intervals in parentheses. Asterisks denote significant differences from the no substrate mean at *;

*P*<0.05, **; *P*<0.01 and ***; *P*<0.001.

**Supplementary Table 2** | Indicator species identified for unamended soils and those amended with glucose, glycine and TSB, based on data from 2009–2012. OTUs among the 30 most frequent taxa in the dataset are shaded.

| Indicator group | OTU | Kingdom | Phylum | Class | Order | Family | Genus and species | Occupancy* | Statistic | *P* value |
| --- | --- | --- | --- | --- | --- | --- | --- | --- | --- | --- |
| Unamended | OTU_11 | Fungi | Basidiomycota | Microbotryomycetes | Leucosporidiales | Leucosporidiaceae | *Leucosporidium* sp. | 94 | 0.608 | 0.001 |
| Unamended | OTU_115 | Fungi | Ascomycota | Eurotiomycetes | Chaetothyriales | Herpotrichiellaceae | *Rhinocladiella* sp. | 69 | 0.506 | 0.003 |
| Unamended | OTU_117 | Fungi | Ascomycota | NA | NA | NA | NA | 75 | 0.521 | 0.001 |
| Unamended | OTU_120 | Fungi | NA | NA | NA | NA | NA | 62 | 0.543 | 0.001 |
| Unamended | OTU_124 | Fungi | Ascomycota | NA | NA | NA | NA | 92 | 0.644 | 0.001 |
| Unamended | OTU_134 | Fungi | Ascomycota | Lecanoromycetes | Lecanorales | Lecanoraceae | *Lecanora* sp. | 49 | 0.499 | 0.001 |
| Unamended | OTU_136 | Fungi | Basidiomycota | Tremellomycetes | Tremellales | Bulleribasidiaceae | *Dioszegia antarctica* | 49 | 0.519 | 0.001 |
| Unamended | OTU_141 | Fungi | Basidiomycota | NA | NA | NA | NA | 23 | 0.485 | 0.001 |
| Unamended | OTU_142 | Fungi | Ascomycota | Eurotiomycetes | Eurotiales | Aspergillaceae | *Penicillium* sp. | 35 | 0.464 | 0.001 |
| Unamended | OTU_143 | Fungi | Ascomycota | Dothideomycetes | Capnodiales | Teratosphaeriaceae | *Elasticomyces elasticus* | 7 | 0.265 | 0.008 |
| Unamended | OTU_145 | Fungi | NA | NA | NA | NA | NA | 27 | 0.427 | 0.001 |
| Unamended | OTU_153 | Fungi | Ascomycota | NA | NA | NA | NA | 19 | 0.419 | 0.001 |
| Unamended | OTU_155 | Fungi | NA | NA | NA | NA | NA | 16 | 0.35 | 0.001 |
| Unamended | OTU_156 | Fungi | Ascomycota | NA | NA | NA | NA | 38 | 0.411 | 0.001 |
| Unamended | OTU_157 | Fungi | Ascomycota | NA | NA | NA | NA | 18 | 0.308 | 0.004 |
| Unamended | OTU_163 | Fungi | NA | NA | NA | NA | NA | 18 | 0.304 | 0.009 |
| Unamended | OTU_179 | Fungi | Ascomycota | Eurotiomycetes | NA | NA | NA | 29 | 0.407 | 0.001 |
| Unamended | OTU_182 | Fungi | Ascomycota | Eurotiomycetes | Verrucariales | Verrucariaceae | *Verrucaria* sp. | 7 | 0.237 | 0.021 |
| Unamended | OTU_198 | Fungi | Basidiomycota | Tremellomycetes | Tremellales | Bulleribasidiaceae | *Dioszegia antarctica* | 28 | 0.332 | 0.006 |
| Unamended | OTU_211 | Fungi | Ascomycota | Eurotiomycetes | Verrucariales | Verrucariaceae | NA | 9 | 0.26 | 0.009 |
| Unamended | OTU_220 | Fungi | NA | NA | NA | NA | NA | 7 | 0.226 | 0.015 |
| Unamended | OTU_222 | Fungi | Ascomycota | NA | NA | NA | NA | 11 | 0.325 | 0.001 |
| Unamended | OTU_213 | Fungi | Ascomycota | Leotiomycetes | Thelebolales | Pseudeurotiaceae | *Pseudogymnoascus* sp. | 10 | 0.323 | 0.001 |
| Unamended | OTU_233 | Fungi | NA | NA | NA | NA | NA | 7 | 0.227 | 0.030 |
| Unamended | OTU_246 | Fungi | Basidiomycota | Malasseziomycetes | Malasseziales | Malasseziaceae | *Malassezia sympodialis* | 11 | 0.304 | 0.002 |
| Unamended | OTU_282 | Fungi | Ascomycota | NA | NA | NA | NA | 10 | 0.283 | 0.003 |
| Unamended | OTU_304 | Fungi | NA | NA | NA | NA | NA | 8 | 0.257 | 0.002 |
|  |  |  |  |  |  |  |  |  |  |  |
| Glucose | OTU_126 | Fungi | Ascomycota | NA | NA | NA | NA | 78 | 0.781 | 0.001 |
| Glucose | OTU_133 | Fungi | Ascomycota | Leotiomycetes | Thelebolales | Pseudeurotiaceae | *Pseudogymnoascus* sp. | 27 | 0.325 | 0.02 |
| Glucose | OTU_162 | Fungi | Ascomycota | Eurotiomycetes | Verrucariales | Verrucariaceae | NA | 44 | 0.695 | 0.001 |
| Glucose | OTU_17 | Fungi | Ascomycota | Dothideomycetes | Capnodiales | Mycosphaerellaceae | *Mycosphaerella tassiana* | 38 | 0.447 | 0.001 |
| Glucose | OTU_196 | Fungi | Ascomycota | Leotiomycetes | Helotiales | Helotiales_fam_Incertae_sedis | *Cadophora malorum* | 30 | 0.42 | 0.001 |
| Glucose | OTU_199 | Fungi | Ascomycota | Dothideomycetes | Pleosporales | Didymellaceae | *Epicoccum nigrum* | 22 | 0.518 | 0.001 |
| Glucose | OTU_253 | Fungi | Basidiomycota | Microbotryomycetes | NA | NA | NA | 10 | 0.254 | 0.008 |
| Glucose | OTU_292 | Fungi | Ascomycota | Eurotiomycetes | Verrucariales | Verrucariaceae | *Verrucaria* sp. | 7 | 0.292 | 0.001 |
| Glucose | OTU_334 | Fungi | NA | NA | NA | NA | NA | 8 | 0.312 | 0.001 |
|  |  |  |  |  |  |  |  |  |  |  |
| Glycine | OTU_189 | Fungi | Ascomycota | Eurotiomycetes | Verrucariales | Verrucariaceae | NA | 15 | 0.332 | 0.001 |
| Glycine | OTU_216 | Fungi | Ascomycota | Eurotiomycetes | NA | NA | NA | 9 | 0.254 | 0.005 |
| Glycine | OTU_227 | Fungi | NA | NA | NA | NA | NA | 10 | 0.228 | 0.036 |
|  |  |  |  |  |  |  |  |  |  |  |
| TSB | OTU_105 | Fungi | Basidiomycota | Tremellomycetes | Cystofilobasidiales | Mrakiaceae | *Mrakia frigida* | 195 | 0.878 | 0.001 |
| TSB | OTU_12 | Fungi | Basidiomycota | Tremellomycetes | Tremellales | Bulleribasidiaceae | *Vishniacozyma tephrensis* | 111 | 0.774 | 0.001 |
| TSB | OTU_121 | Fungi | NA | NA | NA | NA | NA | 98 | 0.775 | 0.001 |
| TSB | OTU_129 | Fungi | Ascomycota | NA | NA | NA | NA | 39 | 0.517 | 0.001 |
| TSB | OTU_212 | Fungi | NA | NA | NA | NA | NA | 21 | 0.362 | 0.001 |
| TSB | OTU_272 | Fungi | Ascomycota | Eurotiomycetes | Verrucariales | Verrucariaceae | *Verrucaria* sp. | 10 | 0.331 | 0.001 |

*Note that taxa with occupancies of <7 are not shown. *Abbreviation*: NA, not assignable.

**Supplementary Table 3 |** Summary of indicator species identified for unchambered (no OTC) and chambered (OTC) soils based on data from 2009–2012. OTUs among the 30 most frequent taxa in the dataset are shaded.

| Indicator group | OTU | Kingdom | Phylum | Class | Order | Family | Genus and species | Occupancy* | Statistic | *P* value |
| --- | --- | --- | --- | --- | --- | --- | --- | --- | --- | --- |
| No OTC | OTU_212 | Fungi | NA | NA | NA | NA | NA | 23 | 0.349 | 0.007 |
| No OTC | OTU_292 | Fungi | Ascomycota | Eurotiomycetes | Verrucariales | Verrucariaceae | *Verrucaria* sp. | 8 | 0.216 | 0.047 |
|  |  |  |  |  |  |  |  |  |  |  |
| OTC | OTU_110 | Fungi | Basidiomycota | Tremellomycetes | Tremellales | Bulleribasidiaceae | *Vishniacozyma* sp. | 114 | 0.55 | 0.001 |
| OTC | OTU_118 | Fungi | NA | NA | NA | NA | NA | 66 | 0.547 | 0.001 |
| OTC | OTU_127 | Fungi | NA | NA | NA | NA | NA | 59 | 0.429 | 0.016 |
| OTC | OTU_13 | Fungi | NA | NA | NA | NA | NA | 48 | 0.446 | 0.001 |
| OTC | OTU_142 | Fungi | Ascomycota | Eurotiomycetes | Eurotiales | Aspergillaceae | *Penicillium* sp. | 35 | 0.384 | 0.001 |
| OTC | OTU_150 | Fungi | Ascomycota | Dothideomycetes | Capnodiales | NA | NA | 49 | 0.394 | 0.003 |
| OTC | OTU_165 | Fungi | NA | NA | NA | NA | NA | 10 | 0.259 | 0.004 |
| OTC | OTU_183 | Fungi | Basidiomycota | NA | NA | NA | NA | 9 | 0.243 | 0.004 |
| OTC | OTU_192 | Fungi | NA | NA | NA | NA | NA | 20 | 0.312 | 0.011 |
| OTC | OTU_211 | Fungi | Ascomycota | Eurotiomycetes | Verrucariales | Verrucariaceae | NA | 9 | 0.259 | 0.001 |
| OTC | OTU_227 | Fungi | NA | NA | NA | NA | NA | 10 | 0.244 | 0.006 |

*Note that taxa with occupancies of <7 are not shown. *Abbreviation*: NA, not assignable.


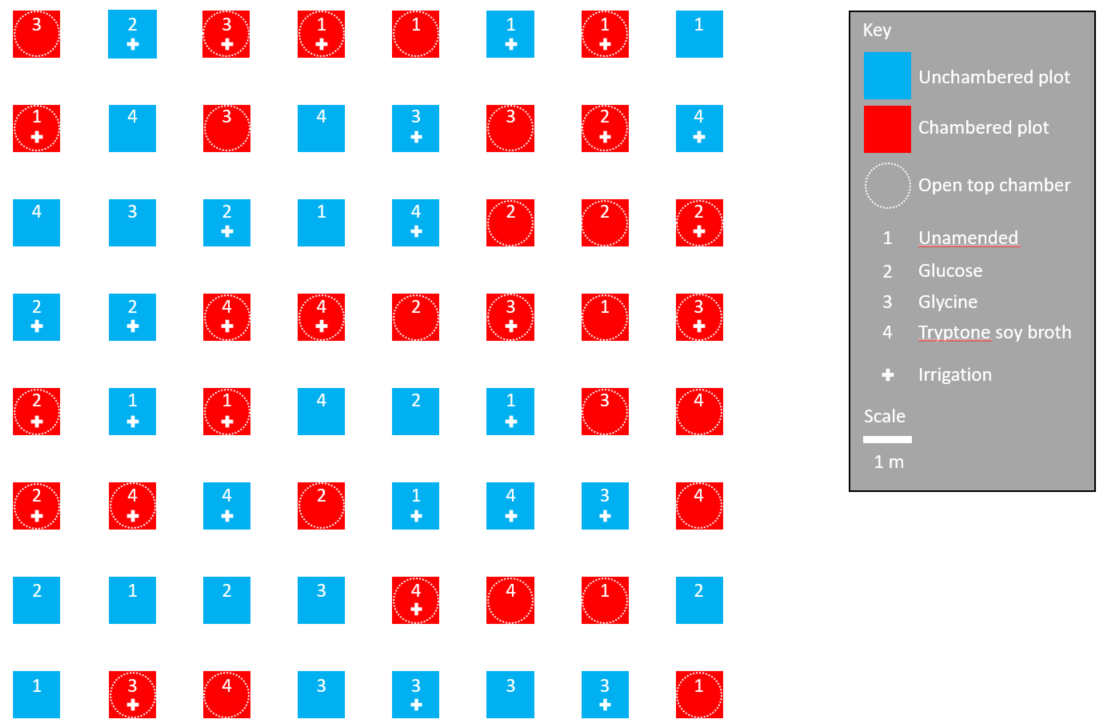


**Supplementary Figure 1 |** Layout of the field experiment at Mars Oasis, which consisted of 64 plots of 1 m^2^ area. Blue and red represent unchambered and chambered plots, respectively, numbers represent substrate treatments and crosses denote irrigation (see key). The experimental layout resulted in 16 OTC-irrigation-substrate treatments, each replicated four times in a randomised design.


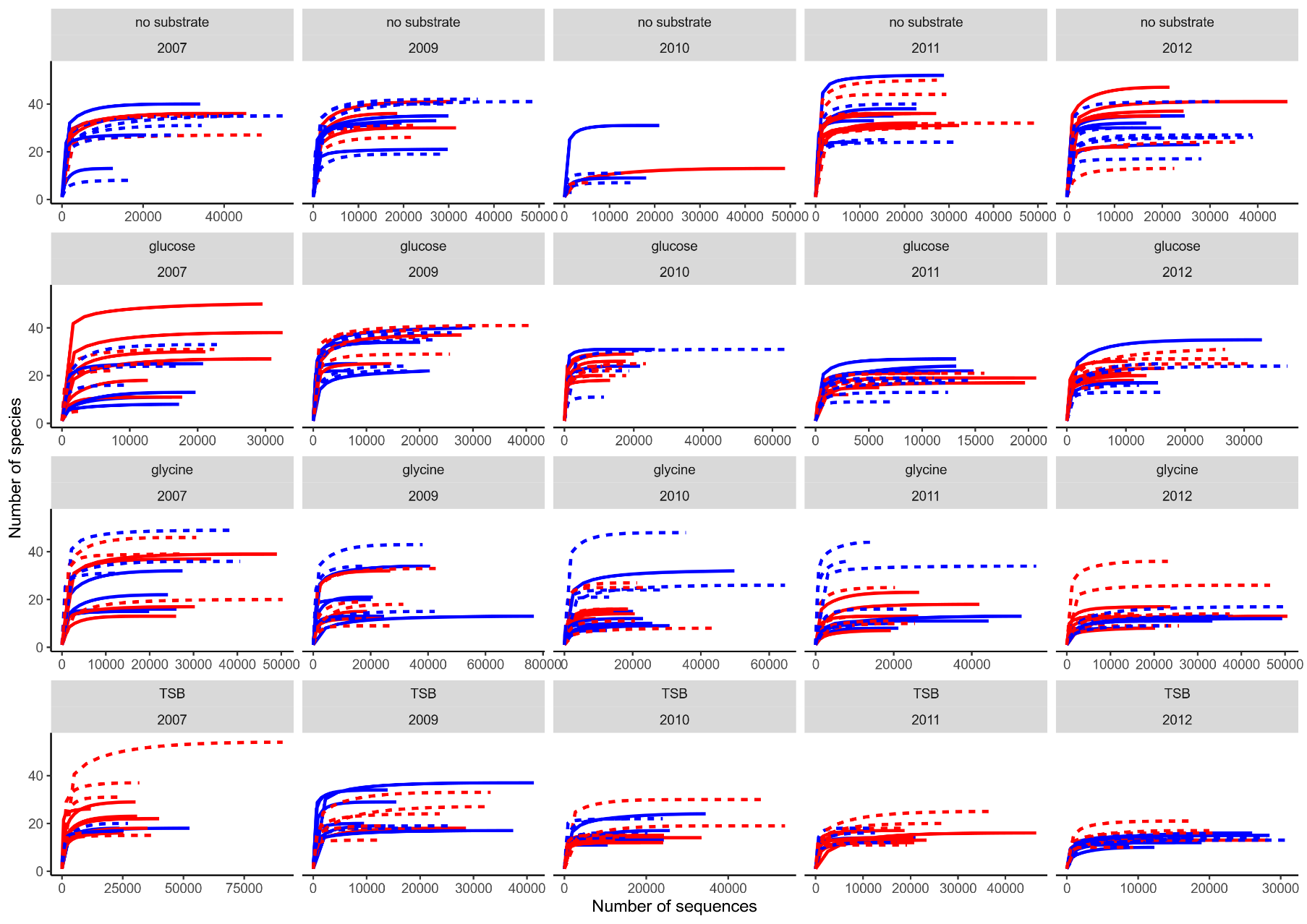


**Supplementary Figure 2 |** OTU accumulation curves by substrate treatment and year. Red and blue lines denote chambered and unchambered plots, and solid and dashed lines denote irrigated and unirrigated plots, respectively.


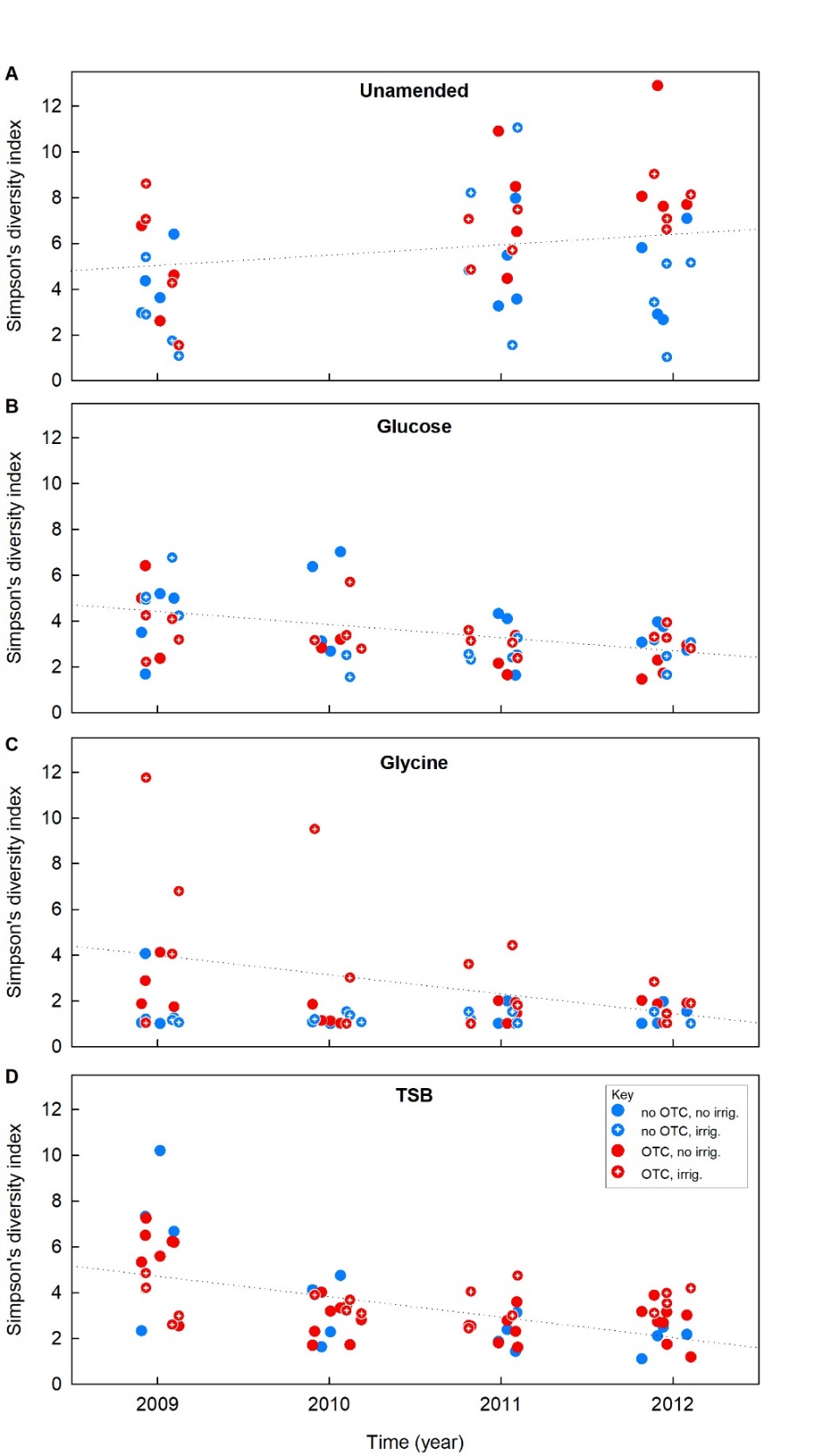


**Supplementary Figure 3 |** Effects of open top chambers, irrigation and substrates on Simpson’s diversity index in **(A)** unamended soils and **(B–D)** glucose-, glycine- and TSB-amended soils in 2009–2012. Dotted lines are significant (*P*<0.05) linear fits. Note that *x*-axis values have been jittered to allow clear visualization of data points and that data in **(A)** for 2010 were deleted owing to low sequencing depth. *Abbreviations*: OTC, open top chamber; irrig., irrigation.


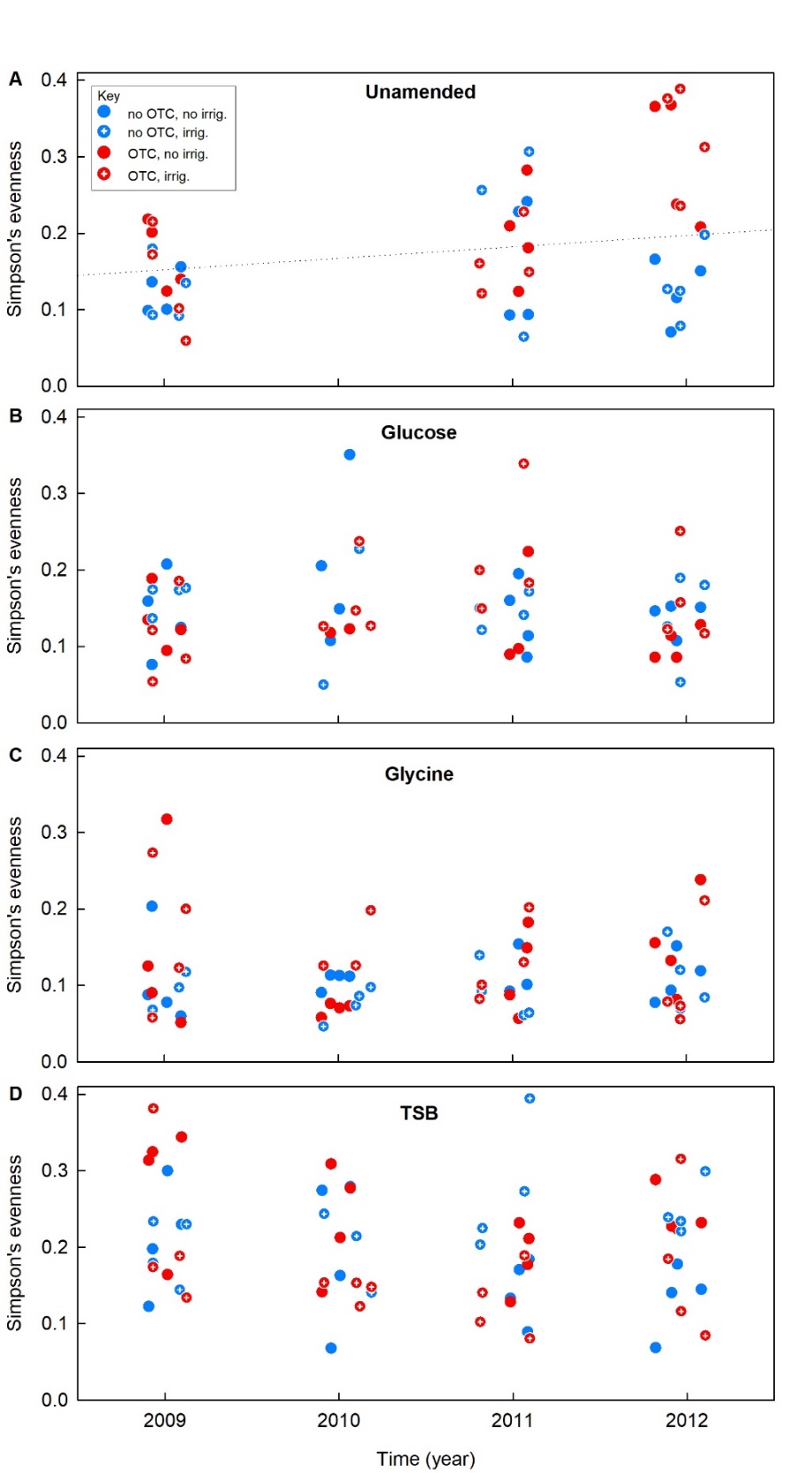


**Supplementary Figure 4 |** Effects of open top chambers, irrigation and substrates on Simpson’s evenness in **(A)** unamended soils and **(B–D)** glucose-, glycine- and TSB-amended soils in 2009–2012. The dotted line in **(A)** is a significant (*P*=0.007) linear fit. Note that *x*-axis values have been jittered to allow clear visualization of data points and that data in **(A)** for 2010 were deleted owing to low sequencing depth. *Abbreviations*: OTC, open top chamber; irrig., irrigation.


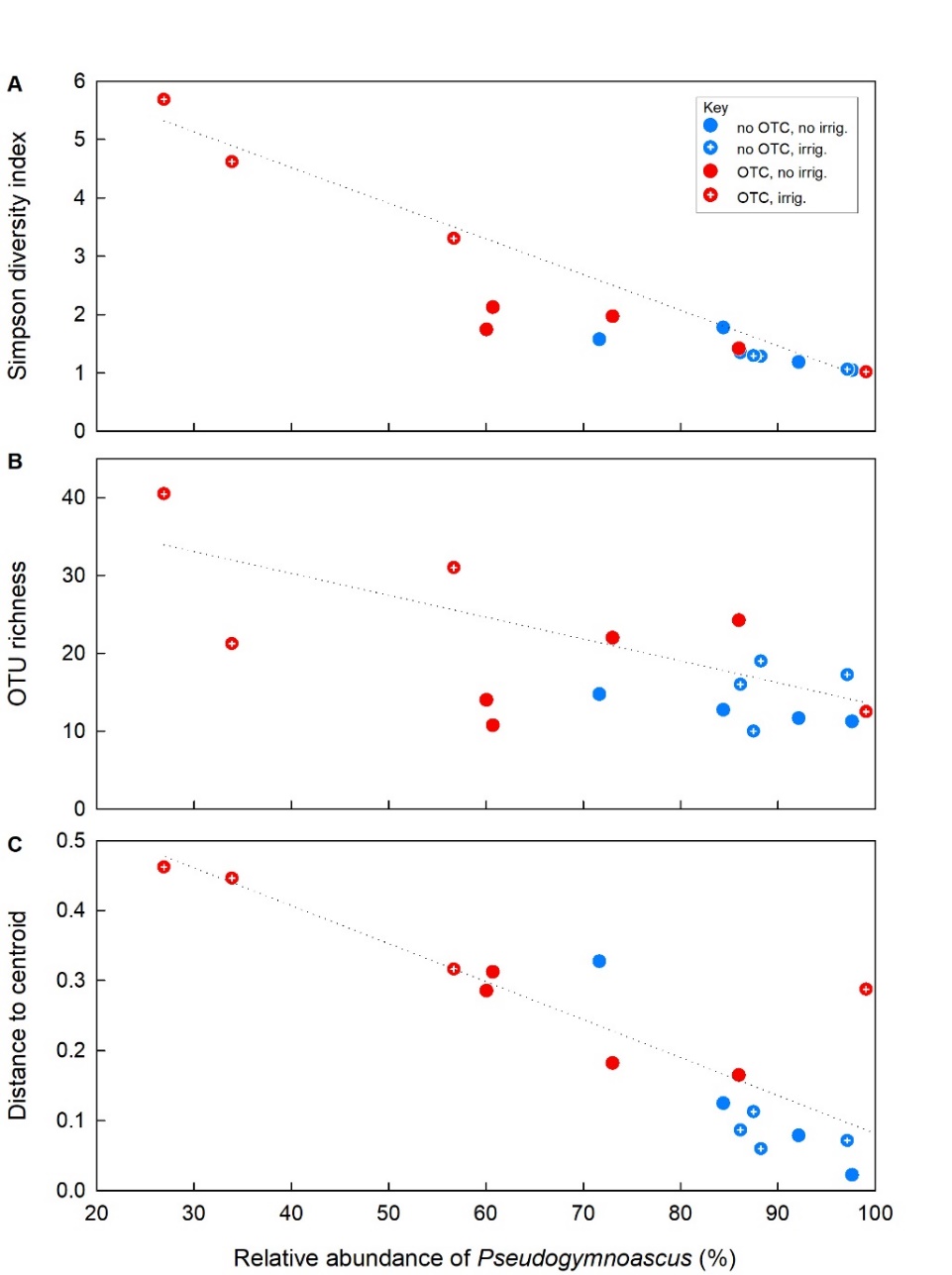


**Supplementary Figure 5 |** **(A)** Simpson’s diversity index, **(B)** OTU richness and **(C)** distance to centroid values as a function of the relative abundance of *Pseudogymnoascus* sp. (OTU 0) in glycine-amended soils in 2009–2012. Dotted lines are significant (*P*≤0.006) linear fits. *Abbreviations*: OTC, open top chamber; irrig., irrigation.


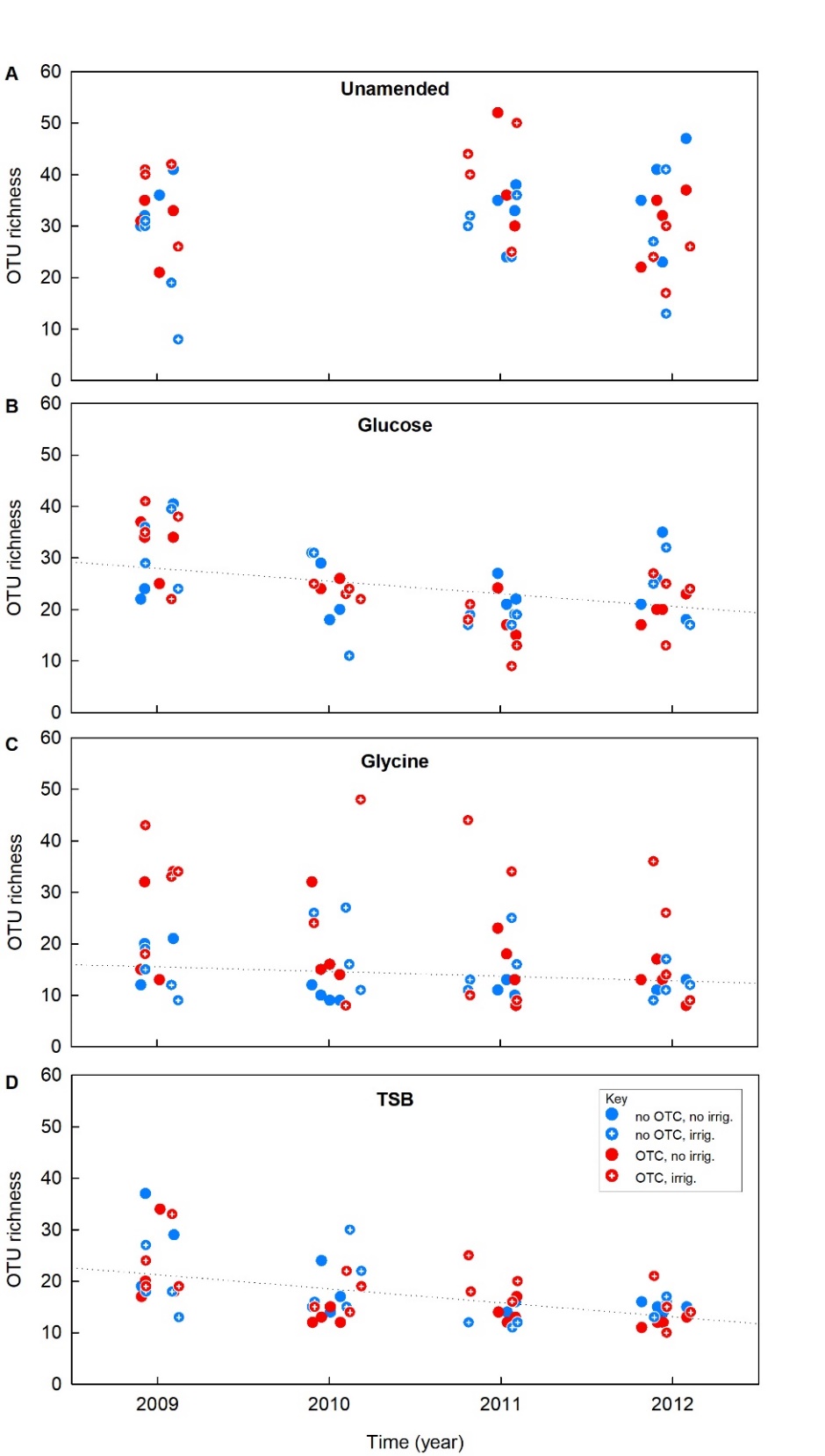


**Supplementary Figure 6 |** Effects of open top chambers, irrigation and substrates on OTU richness in **(A)** unamended soils and **(B–D)** glucose-, glycine- and TSB-amended soils in 2009–2012. Dotted lines are significant (*P*<0.05) linear fits. Note that *x*-axis values have been jittered to allow clear visualization of data points and that data in **(A)** for 2010 were deleted owing to low sequencing depth. *Abbreviations*: OTC, open top chamber; irrig., irrigation.


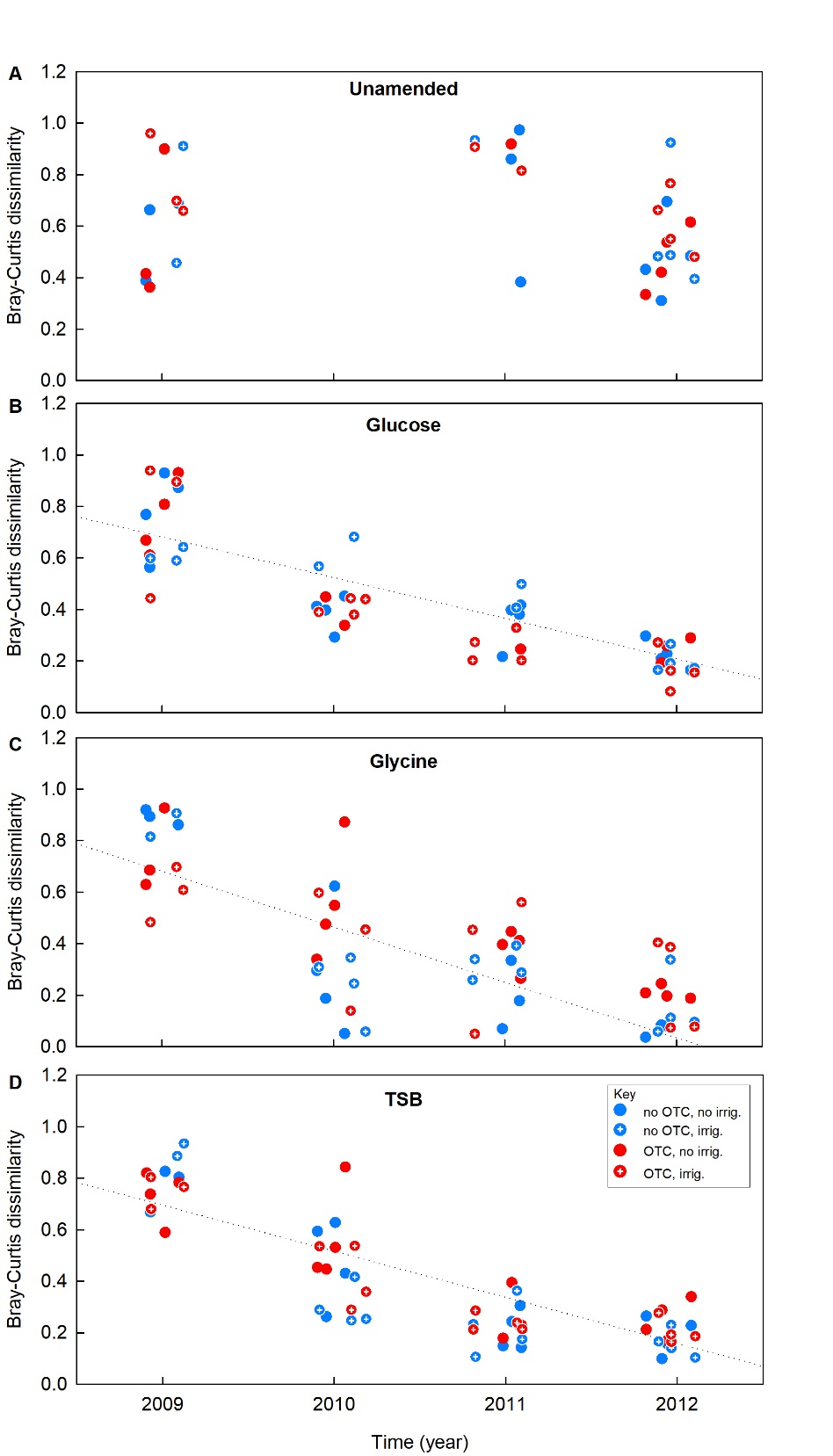


**Supplementary Figure 7 |** Effects of open top chambers, irrigation and substrates on per-plot pairwise Bray-Curtis dissimilarity values over successive years in **(A)** unamended soils and **(B–D)** glucose-, glycine- and TSB-amended soils in 2009–2012. Categories on the *x*-axis represent comparisons for the indicated year and the previous sampling point (e.g., points for 2012 represent per-plot comparisons between 2012 and 2011). Dotted lines are significant (*P*<0.05) linear fits. Note that *x*-axis values have been jittered to allow clear visualization of data points and that data in **(A)** for 2010 were deleted owing to low sequencing depth. *Abbreviations*: OTC, open top chamber; irrig., irrigation.

**
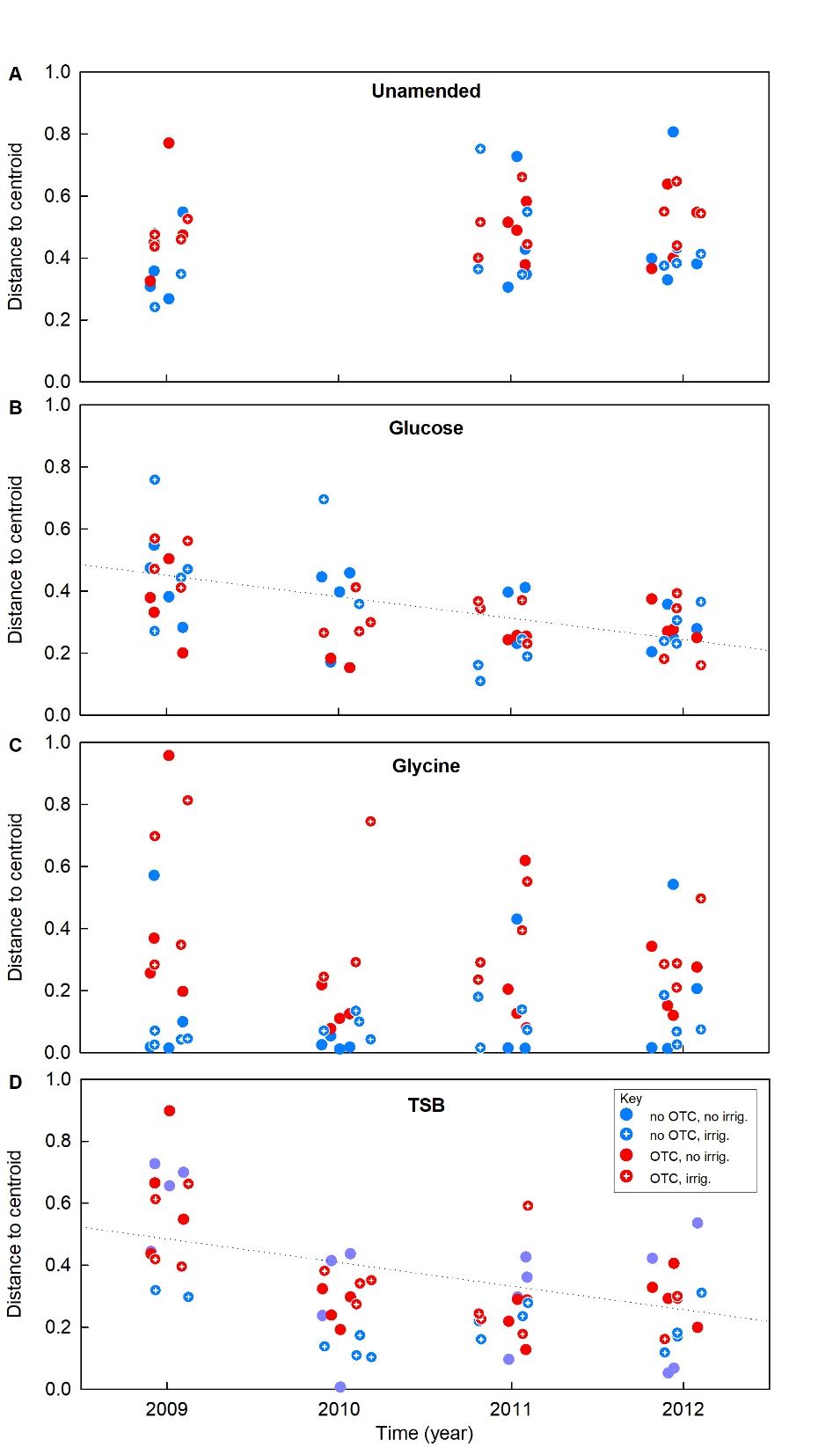
**

**Supplementary Figure 8 |** Effects of open top chambers, irrigation and substrates on distance to centroid values in **(A)** unamended soils and **(B–D)** glucose-, glycine- and TSB-amended soils in 2009–2012. Dotted lines are significant (*P*<0.05) linear fits. Note that *x*-axis values have been jittered to allow clear visualization of data points and that data in **(A)** for 2010 were deleted owing to low sequencing depth. *Abbreviations*: OTC, open top chamber; irrig., irrigation.


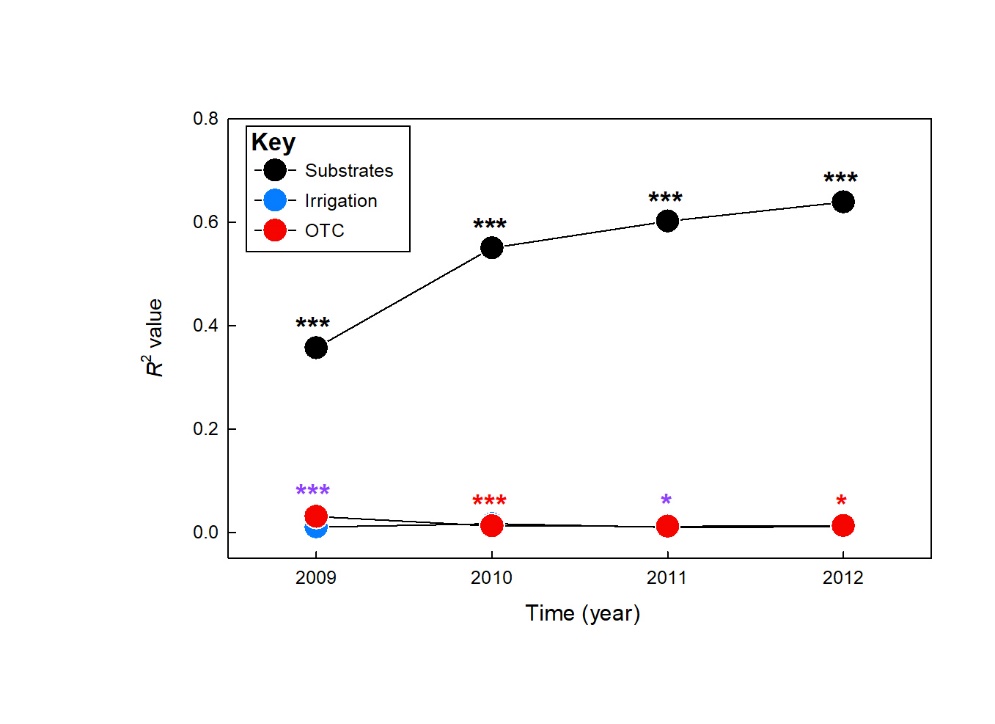


**Supplementary Figure 9 |** The proportion of variation in community composition explained at each sampling in PERMANOVA analyses by substrates, irrigation and OTCs. See key for colour codings of treatments. The asterisks denote the significance of each explanatory variable, with the same colour codings as used for the treatments.


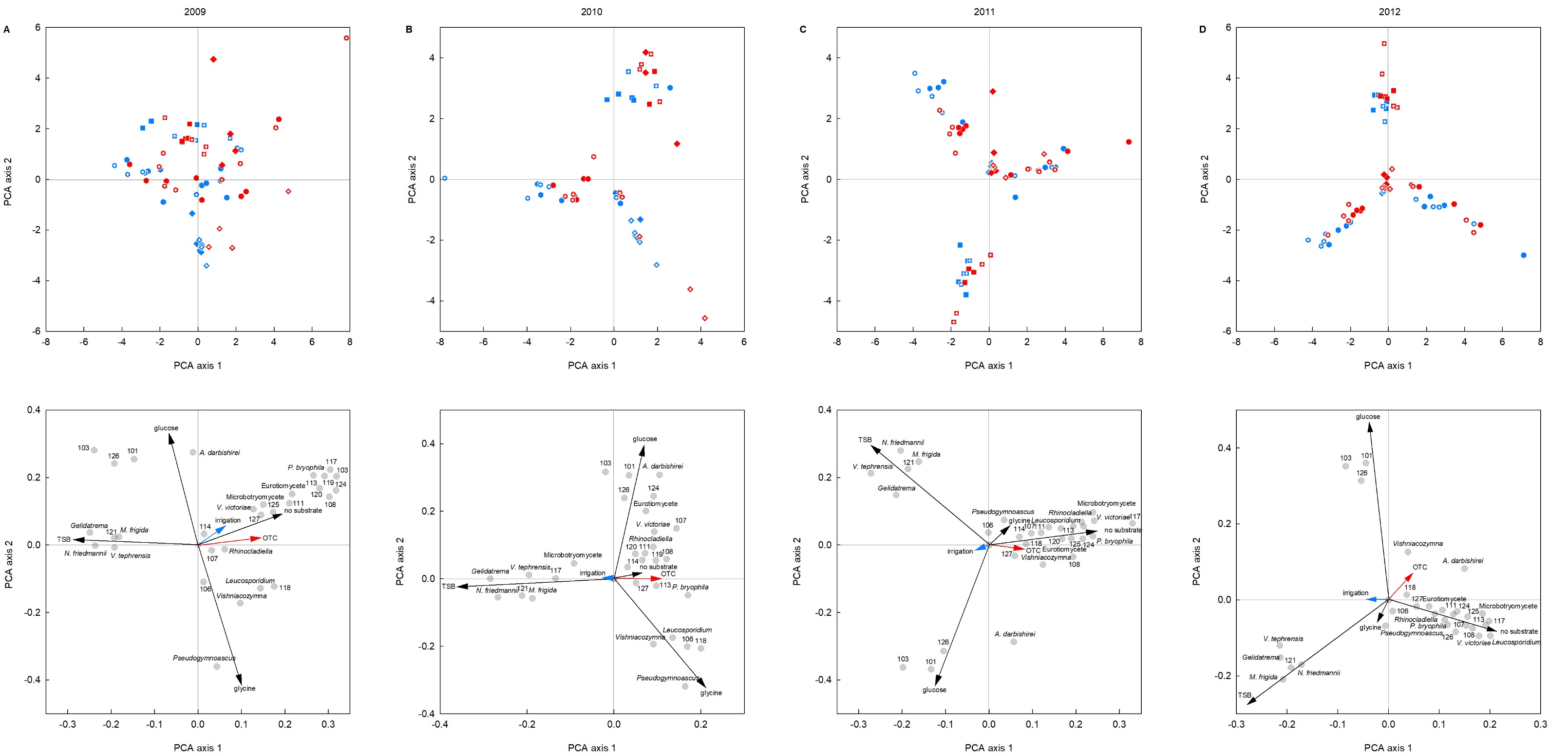


**Supplementary Figure 10 |** Principal components analysis score plots (upper row) and loading plots (lower row) of associations between the relative abundances of the 30 most frequent fungal taxa and treatments in **(A)** 2009, **(B)** 2010, **(C)** 2011 and **(D)** 2012. Blue and red symbols denote unchambered and chambered soils, respectively, and symbols with a cross indicate irrigated soils. Circles, squares, diamonds and hexagons in score plots denote unamended soils and those to which glucose, glycine or TSB were applied, respectively. Red, blue and black arrows in loading plots denote OTC, irrigation and substrate vectors, respectively, and circles show the positions of taxa. Numbers correspond to taxa named at kingdom or phylum level in Table 1.


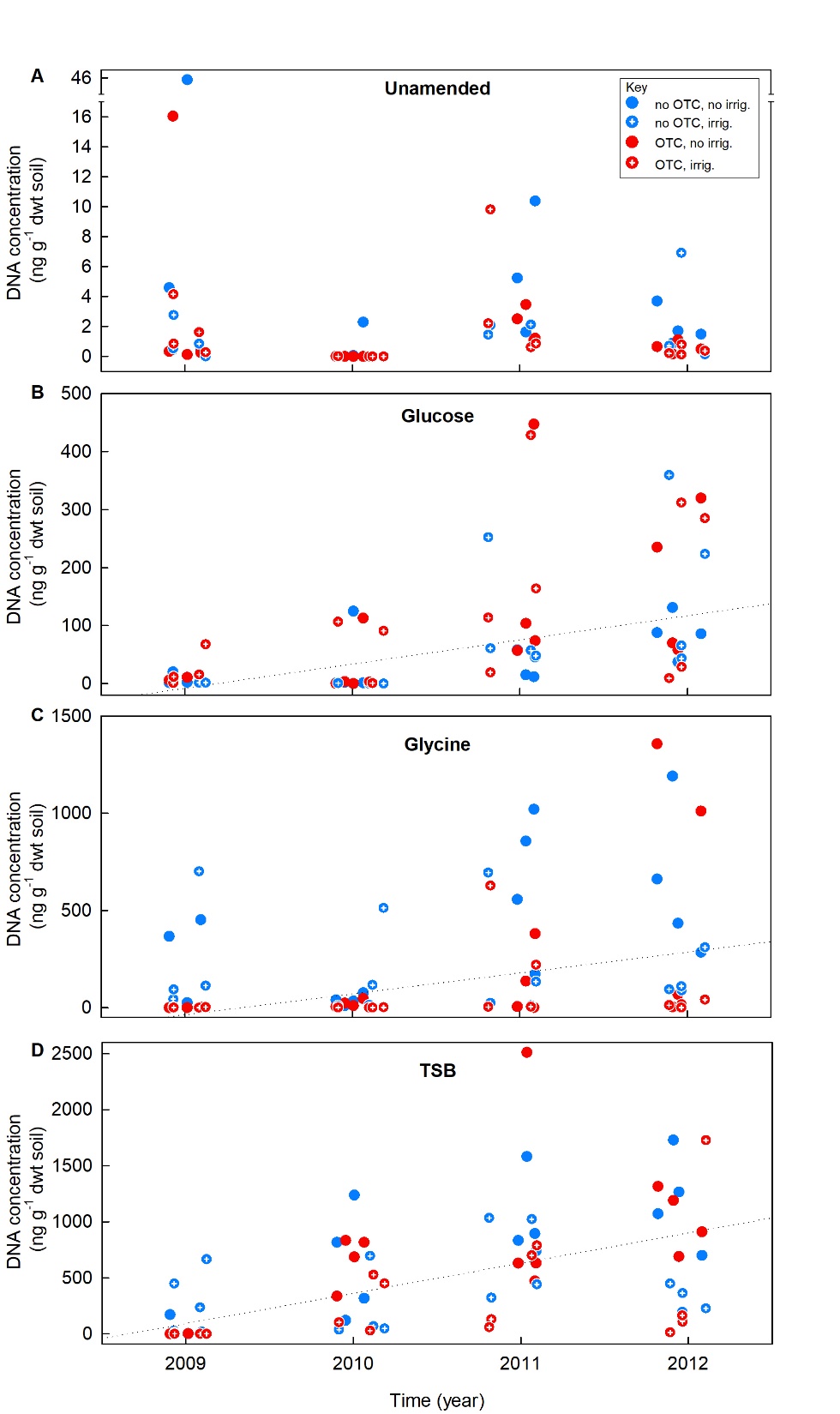


**Supplementary Figure 11 |** Effects of open top chambers, irrigation and substrates on fungal DNA concentrations in **(A)** unamended soils and **(B–D)** glucose-, glycine- and TSB-amended soils in 2009–2012. Dotted lines are significant (*P*<0.05) linear fits. Note the split *y*-axis in **(A)**, that *x*-axis values have been jittered to allow clear visualization of data points, and that *y*-axes are not identically scaled. *Abbreviations*: OTC, open top chamber; irrig., irrigation.
